# Comparative cellular, physiological and transcriptome analyses reveal the potential easy dehulling mechanism of rice-tartary buckwheat (*Fagopyrum Tararicum*)

**DOI:** 10.1186/s12870-020-02715-7

**Published:** 2020-11-04

**Authors:** Hong-You Li, Chao-Xin Wu, Qiu-Yu Lv, Tao-Xiong Shi, Qi-Jiao Chen, Qing-Fu Chen

**Affiliations:** 1 Research Center of Buckwheat Industry Technology, Guizhou Normal University, Guiyang, 550001 China; 2 School of Big Data and Computer Science, Guizhou Normal University, Guiyang, 550025 China

**Keywords:** Rice-tartary buckwheat, Easy dehulling, Hull development, Comparative transcriptome, WGCNA, Cell wall, Cellulose, Hemicellulose, Molecular mechanism

## Abstract

**Background:**

Tartary buckwheat has gained popularity in the food marketplace due to its abundant nutrients and high bioactive flavonoid content. However, its difficult dehulling process has severely restricted its food processing industry development. Rice-tartary buckwheat, a rare local variety, is very easily dehulled, but the cellular, physiological and molecular mechanisms responsible for this easy dehulling remains largely unclear.

**Results:**

In this study, we integrated analyses of the comparative cellular, physiological, transcriptome, and gene coexpression network to insight into the reason that rice-tartary buckwheat is easy to dehull. Compared to normal tartary buckwheat, rice-tartary buckwheat has significantly brittler and thinner hull, and thinner cell wall in hull sclerenchyma cells. Furthermore, the cellulose, hemicellulose, and lignin contents of rice-tartary buckwheat hull were significantly lower than those in all or part of the tested normal tartary buckwheat cultivars, respectively, and the significant difference in cellulose and hemicellulose contents between rice-tartary buckwheat and normal tartary buckwheat began at 10 days after pollination (DAP). Comparative transcriptome analysis identified a total of 9250 differentially expressed genes (DEGs) between the rice- and normal-tartary buckwheat hulls at four different development stages. Weighted gene coexpression network analysis (WGCNA) of all DEGs identified a key module associated with the formation of the hull difference between rice- and normal-tartary buckwheat. In this specific module, many secondary cell wall (SCW) biosynthesis regulatory and structural genes, which involved in cellulose and hemicellulose biosynthesis, were identified as hub genes and displayed coexpression. These identified hub genes of SCW biosynthesis were significantly lower expression in rice-tartary buckwheat hull than in normal tartary buckwheat at the early hull development stages. Among them, the expression of 17 SCW biosynthesis relative-hub genes were further verified by quantitative real-time polymerase chain reaction (qRT-PCR).

**Conclusions:**

Our results showed that the lower expression of SCW biosynthesis regulatory and structural genes in rice-tartary buckwheat hull in the early development stages contributes to its easy dehulling by reducing the content of cell wall chemical components, which further effects the cell wall thickness of hull sclerenchyma cells, and hull thickness and mechanical strength.

**Supplementary Information:**

**Supplementary information** accompanies this paper at 10.1186/s12870-020-02715-7.

## Background

Tartary buckwheat (*Fagopyrum tataricum* Gaertn.), a member of Polygonaceae family, is a pseudo-cereal crop and mainly grows in mountainous areas of western China and the Himalyas [1]. In recent years, tartary buckwheat has attracted worldwide attention and gained popularity in the food marketplace due to its abundant nutrients, especially the higher content of bioactive flavonoids which contribute to diverse human health benefits [2–6]. Seed is the major exploitive part of tartary buckwheat for humans [2], and has been widely used to develop health products such as wine, tea, noodles, cookies, etc. except for direct use. However, the seeds of all normal tartary buckwheat cultivars are tightly surrounded by tough hulls, which are difficult to remove before seeds can be used or processed in foods. Although many methods have been developed to solve tartary buckwheat’s hard dehulling issue, these methods still face many problems such as low dehulling rate (≤35%), broken groats, and nutrient loss (including flavonoids) caused by cooking before dehulling [7–9]. These situations have severely restricted the development of tartary buckwheat food processing industry. Fortunately, rice-tartary buckwheat, a very rare and easily dehulled tartary buckwheat, has been found growing on a very small scale in southern China and the Himalayan hills [10]. Although the rice-tartary buckwheat has very small seeds, low yield, and late maturity, its excellent dehulling property can help in improving normal tartary buckwheat cultivars, which will significantly help in solving the dehulling problem.

Generally, a full understanding of the cellular, physiological, genetic, and molecular mechanisms of an excellent agronomic trait is the crucial premise for utilizing this trait to improve crops. To date, several studies have been performed to investigate the genetic mechanisms that are responsible for the easy dehulling of rice-tartary buckwheat. It has been known that the easy dehulling of rice-tartary buckwheat is a recessive trait that is controlled by a single recessive gene though the use of F_2_ populations of a cross of rice-tartary buckwheat and different normal tartary buckwheat cultivars [11–13]. Currently, some candidate genes that controlled this easy dehulling trait of rice-tartary buckwheat have been identified by RNA sequencing, bulked segregant and gene expression analyses [14–17]. Furthermore, some phenotypic studies found that the hull ditch that located between the two edges of rice-tartary buckwheat seeds is thinner than that of normal tartary buckwheat, which suggested that the thin hull of rice-tartary buckwheat contributed to the easy dehulling, and further defined the easy dehulling trait as the thin hull trait [12, 13, 18]. Recently, one physiological research found that the amounts of hull cellulose and lignin (which are the major chemical components of the cell wall) in normal tartary buckwheat were significantly higher or lower than in rice-tartary buckwheat and common buckwheat (another cultivated buckwheat that is related-easily dehulled) [19–21], respectively. However, there is no obvious difference in the total amount of lignin and cellulose between normal tartary buckwheat and rice-tartary buckwheat [21], suggesting that the ratio of lignin to cellulose in the hull might be a contributing factor in dehulling degree. Considering the major chemical components of the hull also contains hemicellulose besides cellulose and lignin, it is need still to be explored whether the hemicellulose content of the hull also effects the dehull degree. In addition, although studies of the physiological and genetic mechanisms involved in easy dehulling of rice-tartary buckwheat have got much progressed, the cellular and molecular mechanism of easy dehulling remains largely unknown, which limits the knowledge of breeding new easy dehulling tartary buckwheat varieties.

In this study, we carried out comparative cell, physiology, transcriptome, and coexpression network analyses to insight into the cellular, physiological and molecular mechanisms that make rice-tartary buckwheat easy to dehull. Our results revealed the cellular and physiological difference of hull between rice-tartary buckwheat and normal tartary buckwheat, found the key period during which hull differences between rice- and normal-tartary buckwheat are determined, identified the key gene regulatory network that contributed to dehulling ease of tartary buckwheat, and found the molecular mechanism responsible for the easy dehulling of rice-tartary buckwheat. These results not only expanded our understanding of the easy dehulling mechanism of rice-tartary buckwheat, but also provided valuable information for further examining the function of candidate genes governing dehulling in rice-tartary buckwheat and improving the dehulling of normal tartary buckwheat through gene manipulation.

## Results

### Phenotypic and physiological changes

**Fig. 1:**
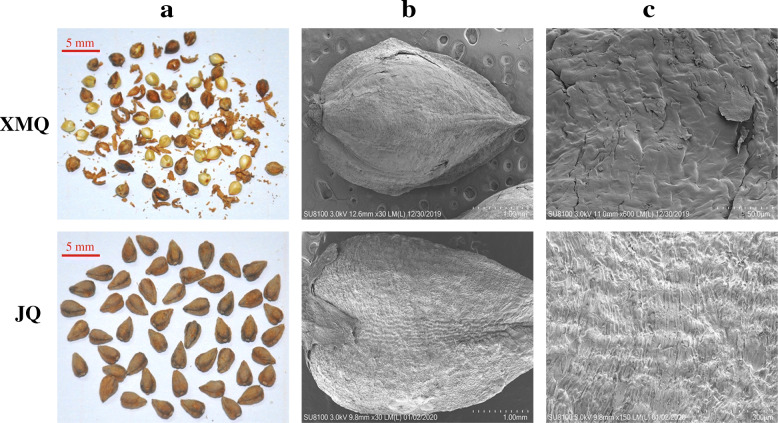
Phenotypes of XMQ and JQ mature seed hulls. **a** Phenotypes of XMQ and JQ mature seed after manual dehulling by rubbing 10 times. **b** Scanning electron micrographs of XMQ and JQ mature seed. **c** Scanning electron micrographs of XMQ and JQ mature seed hull

**Fig. 2:**
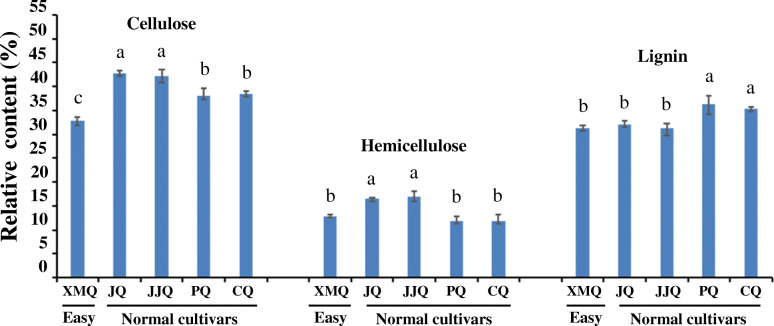
Cellulose, hemicellulose and lignin contents in the seed hull of different tartary buckwheat varieties. Values were the averages of three independent biological replicates, and bars with different letters represented significant differences (*P* < 0.05)

Compared with normal tartary buckwheat cultivar “JQ”, the matured hull of rice-tartary buckwheat “XMQ” showed markedly reduced mechanical strength, which was easily broken and shedding when rubbed by hand (Fig. 1a). Scanning electron microscopy showed that many flaws occurred on the “XMQ” hull that did not exist on the “JQ” (Fig. 1b, c). These results suggested that the lower mechanical strength of the “XMQ” hull contributed to its easy dehulling, and the easy dehulling trait was redefined as “brittle hull” trait. To further investigate whether the lower mechanical strength of the “XMQ” hull was caused by the change of hull compositions, we measured the hull cellulose, hemicellulose, and lignin contents of “XMQ”, “JQ”, and other three normal tartary buckwheat cultivars (“JJQ”, “PQ” and “CQ”). As shown in Fig. 2, when compared with the four normal tartary buckwheat cultivars, the hull cellulose content of “XMQ” was significantly lower than all tested normal tartary buckwheat cultivars, while the hull hemicellulose and lignin contents of “XMQ” were only significantly lower than among two normal tartary buckwheat cultivars. These results suggested that the reduced cellulose, hemicellulose, and lignin contents might contribute to the brittleness and easy dehulling of rice-tartary buckwheat, of which the reduced cellulose content might be the major reason.

### Dynamic change of cellulose and hemicellulose contents, and hull thickness in “XMQ” and “JQ”

**Fig. 3:**
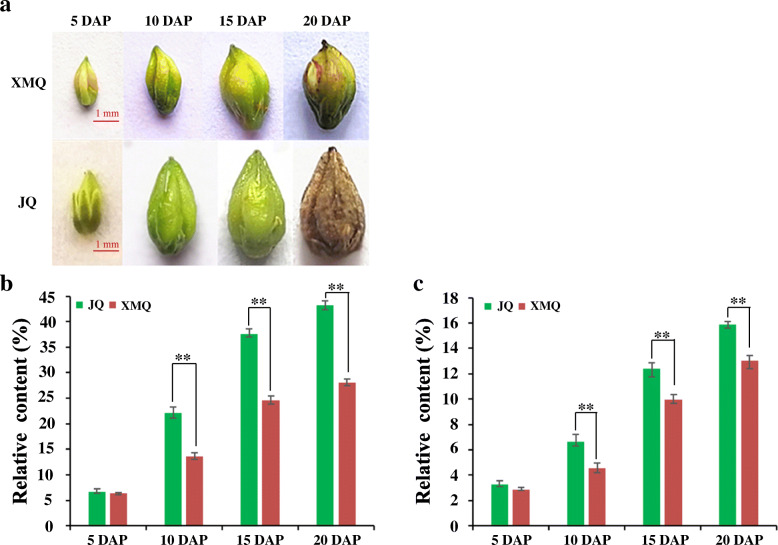
Seed phenotypes (**a**), hull cellulose (**b**) and hemicellulose content (**c**) of XMQ and JQ. Asterisks indicated significant differences (** *P* < 0.01)

**Fig. 4:**
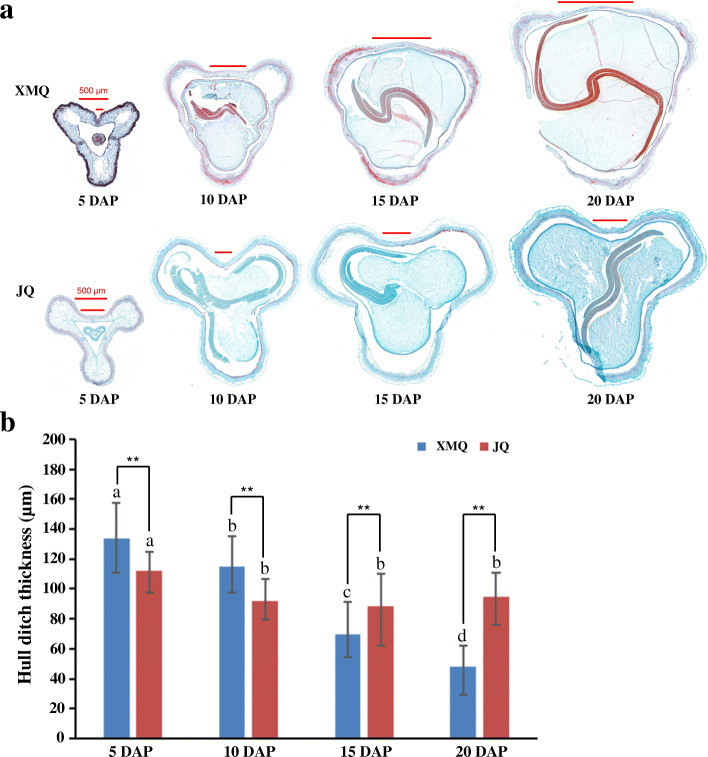
Seed paraffin section micrographs (**a**) and hull ditch thickness (**b**) of XMQ and JQ. Bars with different letters represented significant differences (*P* < 0.05) in the same variety at different development stages. Asterisks indicated significant differences (** *P* < 0.01) between two varieties at the same development stage

**Fig. 5:**
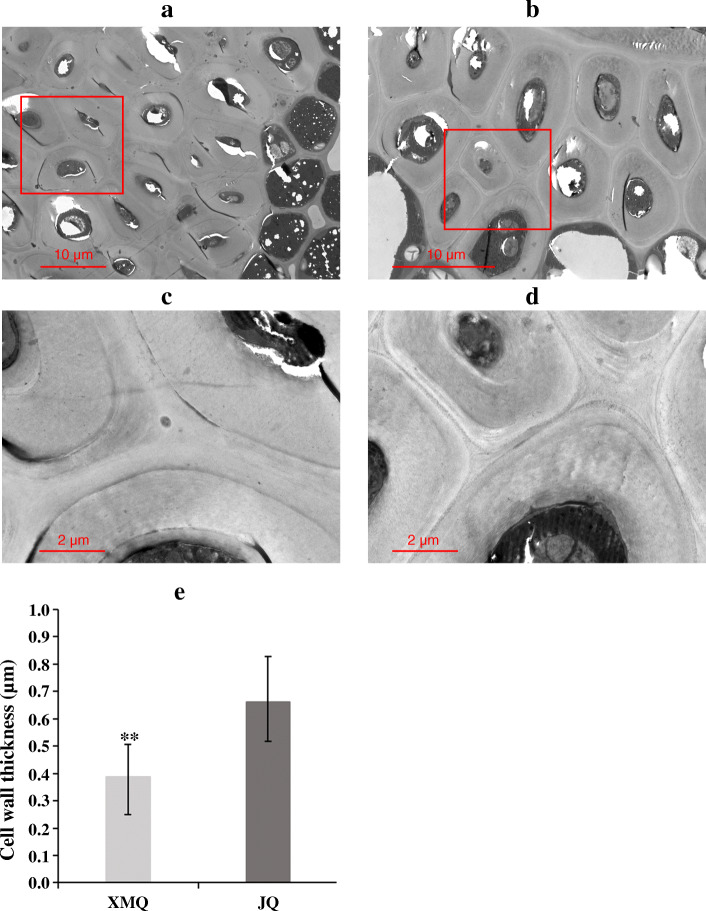
Anatomical features of sclerenchyma cells in the 15 DAP seed hull of JQ and XMQ. **a** and **c** the transmission electron micrographs of sclerenchyma cell walls in JQ. **b** and **d** the transmission electron micrographs of sclerenchyma cell walls in XMQ. **e** the cell wall thickness of sclerenchyma cells in JQ and XMQ. Asterisks indicated significant differences (** *P* < 0.01) between JQ and XMQ

To investigate the accumulation of cellulose and hemicellulose during hull development, the contents of cellulose and hemicellulose in seed hulls of “XMQ” and “JQ” were determined at four different stages (Fig. 3a). As shown in Fig. 3b, c, the two cultivars displayed a similar trend in the change of cellulose and hemicellulose contents, which were sustained growth during hull development. In addition, the significant difference in cellulose and hemicellulose contents between the two cultivars started to occur at 10 DAP. To further insight into whether the hull ditch thickness changes in the two cultivars during seed development, paraffin section analysis was performed and the ditch thickness of the hull was measured according to paraffin section results (Fig. 4). The hull ditch thickness of “XMQ” showed significantly decreased during seed development, while the hull ditch thickness of “JQ” only decreased at 10 DAP compared with 5 DAP and no obvious difference was observed after 10 DAP (Fig. 4b). In addition, the hull ditch thickness of “XMQ” was significantly lower than that of “JQ” at 15 and 20 DAP, although its hull ditch thickness was obviously higher than that of “JQ” at 5 and 10 DAP (Fig. 4b). Transmission electron microscopy further revealed the cell wall thickness of sclerenchyma cells of “XMQ” hull ditch was significantly thinner than “JQ” (Fig. 5). These results indicated that the cellulose content, hemicellulose content, and hull ditch thickness of “XMQ” and “JQ” were dynamic and different changes, and these hull samples could be used for further transcriptome analyses.

### Transcriptome analysis of hull development in tartary buckwheat cultivars

To insight into the transcriptome dynamics during hull development, RNA-Seq analyses of the hull of “XMQ” and “JQ” hull were performed at four different development stages (8 tissues, three independent biological replicates for each tissue, 24 samples in total). A total of 521.39 and 517.72 million clean reads, with an average of 43.43 and 43.09 million clean reads for each sample, were generated for “XMQ” and “JQ”, respectively (Additional file 1: Table S1). 85.84 to 92.74% of clean reads were mapped to the reference genome for each sample, of which 64.54–73.47% were uniquely mapped. A total of 29,843 gene loci, including 28,465 known and 1378 novel gene loci, were generated from all mapped clean reads of 24 samples via Cufflinks and Cuffmerge analyses. PCC between the biological replicates of different tissues changed from 0.90 to 0.99 (except for one replicate of J15 and J20 in “JQ” and one replicate of X15 in “XMQ”, which were filtered for further analyses), indicating the high reliability and reproducibility of the replicates (Additional file 2: Figure S1).

The normalized expression level (FPKM) for each gene was calculated, and genes with FPKM ≥0.1 were considered as expressed. A total of 27,955 genes, including 26,589 known and 1366 novel genes, were expressed in at least one sample. Among them, the number of expressed genes in different tissues changed from 23,575 (X20) to 25,014 (X10) in “XMQ”, and 23,282 (J20) to 24,266 (J5) in “JQ” (Additional file 2: Figure S2a). The proportions of genes presenting very high (FPKM ≥100), high (50 ≤ FPKM < 100), moderate (10 ≤ FPKM < 50), low (2 ≤ FPKM < 10), and very low (0.1 ≤ FPKM < 2) expression were relatively similar in all tissues except in X20 tissue (Additional file 2: Figure S2b). In addition, the number of very-low-expression (about 42%) and moderate-expression genes (about 30%) accounted for the two largest proportions in all tissues (Additional file 2: Figure S2b). Taken together, these analyses indicated that we obtained sufficient coverage of the transcriptome of the development hull in these two tartary buckwheat cultivars.

### Transcriptome comparison of hull development in tartary buckwheat cultivars revealed the critical stages for hull development difference

To investigate the relationships between the hull development transcriptomes from the “XMQ” and “JQ”, HCA (Additional file 2: Figure S3a) and PCA (Additional file 2: Figure S3b) were performed based on the average FPKM values for the 27,955 genes that were expressed in at least one of the eight tissue samples. As results, a higher correlation of the same development stage between the two cultivars was observed except at 20 DAP (Figure S3a). PCA analysis showed X5 and J5 were grouped together, while clear separations were observed between X10 and J10, X15 and J15, and X20 and J20 (Additional file 2: Figure S3b), suggesting that a higher degree of similarity in transcriptional programs between these two cultivars at 5 DAP and obvious transcriptional differences existed at 10, 15, and 20 DAP. In addition, these analyses also indicated that 10 DAP might be the critical stage to determine the hull difference formation between the two cultivars at the molecular level.

### Identification of genes that specifically/preferentially expressed in each stage of hull development in both the cultivars

**Fig. 6:**
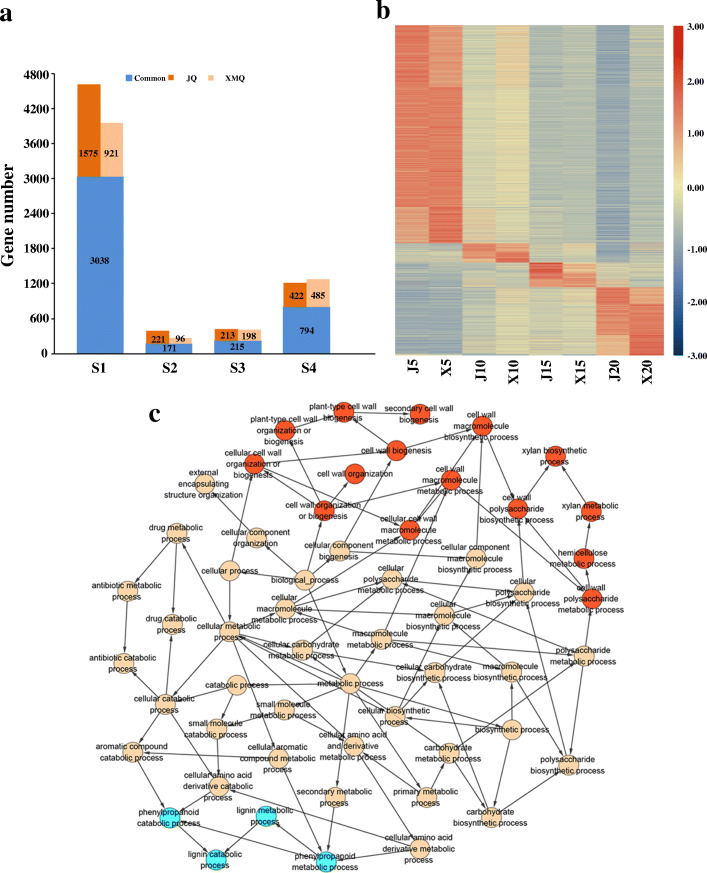
Preferential/stage-specific expression of genes during hull development in XMQ and JQ. **a** The number of specific and common preferentially expressed genes in XMQ and JQ at each development stage. **b** The expression heatmap of preferentially expressed genes in different development stages in XMQ and JQ. **c** GO enrichment map (biological process) of preferentially expressed genes at 10 DAP of hull development in XMQ and JQ

The genes that are specifically/preferentially expressed in each stage of hull development in both cultivars were identified based on the SS algorithm with SS score ≥ 0.5. A total of 6649 and 5918 specific/preferential genes were identified in all four stages for “JQ” and “XMQ”, respectively. Among these genes, 348 (5.2%) and 302 (5.1%) specific/preferential genes were encoded for transcription factors (TFs). The number of stage-specific/preferential genes ranged from 392 to 4613 for “JQ” and 267 to 3959 for “XMQ” (Fig. 6a). The 5 DAP and 10 DAP had the largest and lowest of number stage-specific/preferential genes, respectively, for the two cultivars (Fig. 6a). Furthermore, a high proportion of stage-specific/preferential genes was common in both cultivars, and cultivar-specific genes were also observed in all four stages of the two cultivars (Fig. 6a). A heatmap of all these stage-specific/preferentially expressed genes in the two tartary buckwheat cultivars was shown in Fig. 6b. These results indicated that each hull development stage had its own independent and common developmental programs for both cultivars and also accurately reflected the accumulation of endogenous mRNAs in the hull development of the two cultivars.

The gene ontology (GO) enrichment analyses were carried out for the stage-specific/preferential genes of the two cultivars at each stage. The results of biological process enrichment showed that the 5 DAP of hull development was marked by cell cycle and cell biosynthetic process (Additional file 2: Figure S4), the 10 DAP was significantly and specifically marked by cell wall and phenylpropanoid metabolic process (Fig. 6c), the 15 DAP was majorly marked by “sulfur metabolic” (Additional file 2: Figure S5), and the 20 DAP major was involved in lipid metabolic, response biotic and abiotic stress and transport (Additional file 2: Figure S6). These results suggested that these stage-specific/preferential genes performed stage-specific functions during hull development of tartary buckwheat and 10 DAP was the key stage in the determination cell wall biogenesis of the tartary buckwheat hull.

### DGEs between the two tartary buckwheat cultivars

**Fig. 7:**
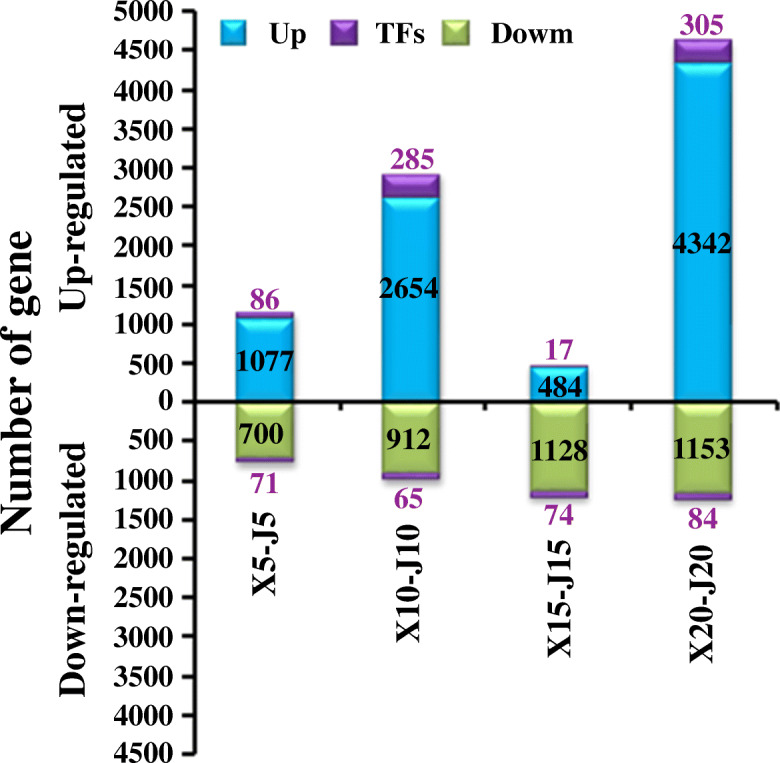
Different expression genes in XMQ vs. JQ at different stages of hull development. The purple number represented the number of up-regulated and down-regulated TFs

Genes that had significantly different expression between “XMQ” and “JQ” were identified at each stage of hull development. A total of 9250 (including 693 TF-encoding genes) and 4187 (294 TF-encoding genes) genes showed significantly higher and lower expression at different stages of hull development in “XMQ” compared with “JQ”, respectively (Fig. 7). Between the two cultivars, the two largest number of DEGs occurred at 20 DAP (5884) and 10 DAP (3916) (Fig. 7), while the fewest occurred at 15 DAP. In addition, some members of most TF families shown significantly different expression in “XMQ”, and the number of different TF families showed an obvious difference, which implied they had diverse functions during hull development (Additional file 2: Figure S7).

To further investigate the biological functions of these DEGs between “XMQ” and “JQ”, GO enrichment analyses were performed. A total of 159, 250, 92 and 161 biological processes were significantly enriched (*P* < 0.05) at 5 DAP, 10 DAP, 15 DAP, and 20 DAP, respectively (Additional file 1: Table S2). Among them, some biological processes were specially/commonly overrepresented at different stages of hull development (Additional file 1: Table S2). Notably, GO terms such as the plant-type primary cell wall biogenesis (GO:0009833), cellulose metabolic process (GO:0030243), cellulose biosynthetic process (GO:0030244), and cellulose catabolic process (GO:0030245) were uniquely enriched at 5 DAP, whereas the hemicellulose metabolic process (GO:0010410) and cell wall modification (GO:0042545) were specially enriched at 10 DAP (Additional file 1: Table S2). In addition, cell wall biogenesis (GO:0042546) and fruit dehiscence (GO:0010047) were significantly enriched at 5 DAP and 10 DAP (Additional file 1: Table S2). These results indicate that 5 DAP and 10 DAP were the key stages that determined the difference in the hull between the two tartary buckwheat cultivars.

### Identification and valuation of the key genes involved in the hull difference between the two tartary buckwheat bultivars by gene coexpression analysis

**Fig. 8:**
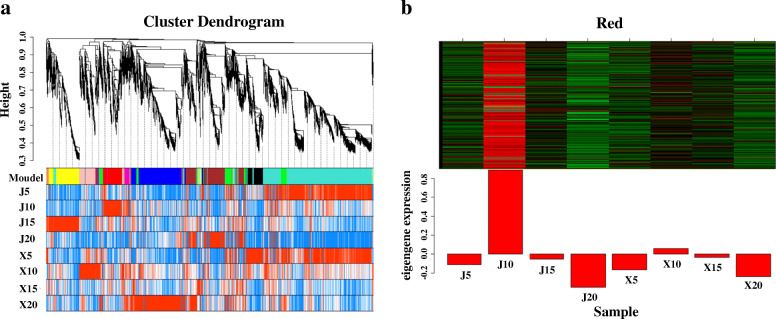
Co-expression network during hull development in XMQ and JQ. **a** Hierarchical clustering tree (dendrogram) of all different expression genes from XMQ vs. JQ based on co-expression network analysis. **b** Identified MEred module with specific high expression genes in JQ hull at 10 DAP

**Fig. 9:**
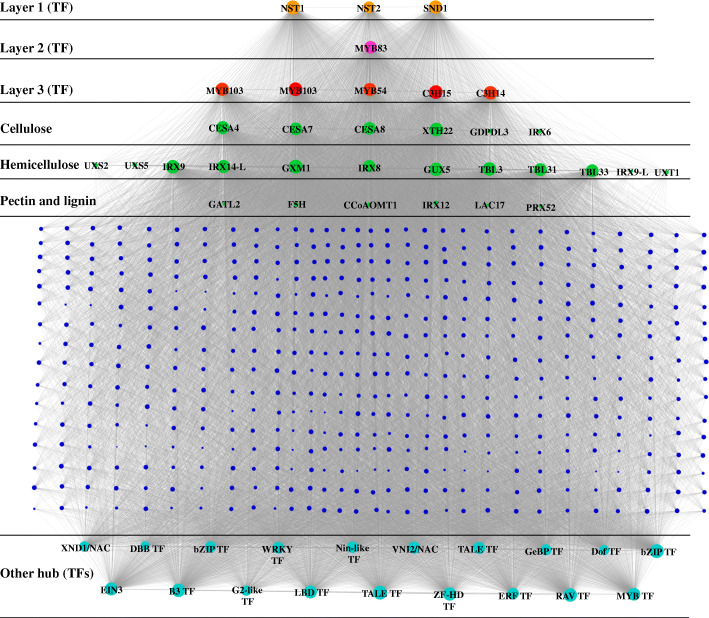
Co-expression network of genes from MEred module. The yellow, pink, and red nodes represented the identified first-, second-, and third-layer regulators of SCW biosynthesis, respectively. Green nodes represented the identified structural genes of SCW biosynthesis. The peacock blue nodes represented the other identified hub TFs. The blue nodes represented other genes. The bigger nodes indicated the hub genes, which had highly connected nodes in this module

To investigate the gene regulatory network (GRN) during hull development and identify the key genes involved in the hull difference between “XMQ” and “JQ”, 9549 genes that were differentially expressed in at least one hull development stage between the two cultivars were used to carry out WGCNA. A total of 18 modules (consisting of 34–3317 genes) were identified and labeled with different colors (Fig. 8a). The correlation analyses of these modules with the cellulose and hemicellulose contents revealed that the MEbrown module presented higher correlation with cellulose content (r = 0.8, *p* = 0.02) (Additional file 2: Figure S8). In this module, genes had specific high expression in the “JQ” hull at 20 DAP. No gene in this module was found to be involved in cellulose and cell wall biosynthesis based on homologous annotation in the *Arabidopsis* TAIR database, which might be caused by the visible hull difference between the two cultivars having formed at 20 DAP. Considering that (1) easy dehulling is a recessive trait, (2) hard dehulling is the corresponding dominant trait, and (3) 10 DAP was identified as the key stage for hull difference formation between the two cultivars, we assumed that the module with genes that presented specific high expression in the “JQ” at 10 DAP was the key module involved in the different hull formation of the two cultivars. As a result, the MEred module was found to meet these standards, which consisted of 533 genes (Fig. 8b). Based on a homologous annotation in the *Arabidopsis* TAIR database, 28 TFs were identified in this module, which represented 17 TF families. All 28 TFs were further identified as hub TFs due to the highly connected nodes in this module. These hub TFs included homologs of *A. thaliana* TFs with known functions of regulating SCW biosynthesis, such as the first-layer NAC regulators (*NST1*, *NST2*, and *NST3*/*SND1*), the second-layer regulator (*MYB46*/*MYB83*), and the third-layer regulators (*MYB54*, *MYB103*, *C3H14*, and *C3H15*) (Additional file 1: Table S3). The homologs of *XND1* and *VNI2*, two NAC TFs that regulate xylem vessel formation, were also identified in these hub TFs (Fig. 9, Additional file 1: Table S4). In addition, several other hub TFs were found to be homologous with *A. thaliana* TFs, which were involved in ethylene signaling (*EIN3*, *ERF71*, and *RAV*), gibberellin biosynthesis (*ATH1*), jasmonic acid signaling (*WRKY50*), and multiple hormonal responses (*MIF2*). Notably, 6, 12, 1, and 5 SCW biosynthesis enzyme genes were also identified in this module (Fig. 9, Additional file 1: Table S3), which were homologs of known downstream targets of *A. thaliana* SCW biosynthesis TFs (NAC and MYB) and catalyzed cellulose biosynthesis, hemicellulose biosynthesis, pectin biosynthesis, and lignin biosynthesis, respectively. Among these enzyme genes, four cellulose biosynthesis genes (*CESA4*, *CESA7*, *CESA8*, and *XTH22*) and eight hemicellulose biosynthesis genes (*IRX8*, *IRX9*, *IRX14-L*, *GXM1*, *GUX5*, *TBL3*, *TBL31* and *TBL33*) were also identified as hub genes in this module (Fig. 9). By comparison, no pectin and lignin biosynthesis enzyme genes were identified as hub genes in this module (Fig. 9).

**Fig. 10:**
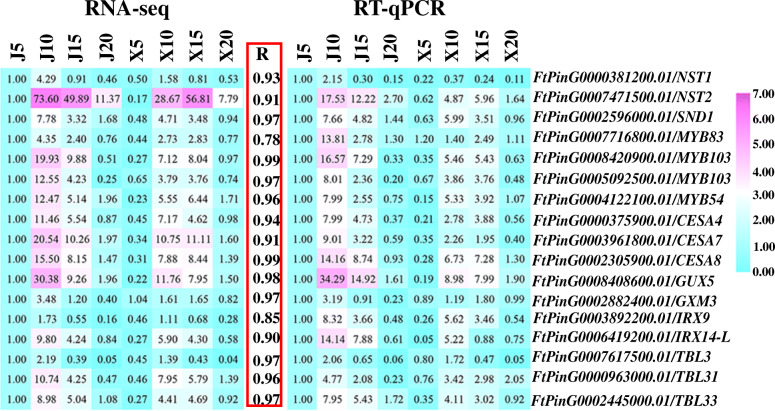
Correlation between expression profiles of the identified SCW biosynthesis genes. The expression fold change of genes was used to construct the heatmaps. The J5 was used as control. The left heatmap represents the RNA-seq, and the right heatmap represents RT-qPCR

**Fig. 11:**
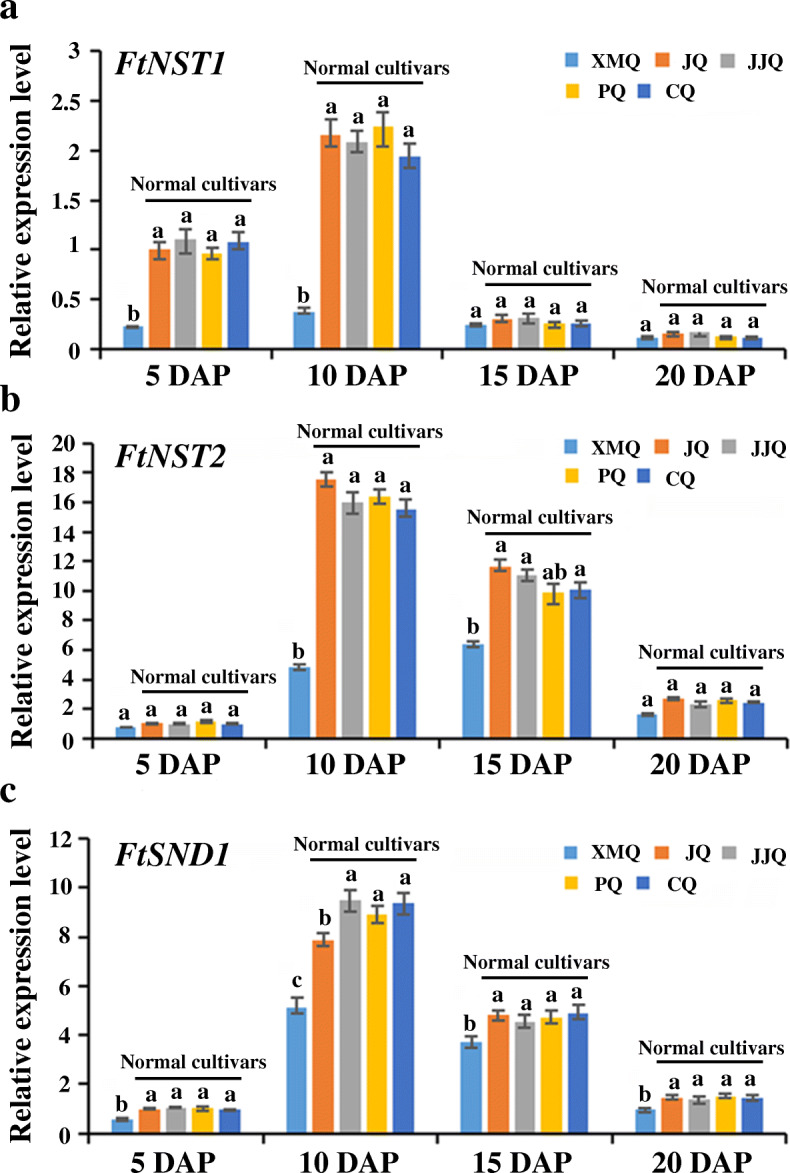
The expression of the first-layer regulatory genes of SCW biosynthesis in different tartary buckwheat cultivars hulls

The expression of most identified hub TF genes and enzyme genes of SCW biosynthesis was significantly higher in “JQ” than in “XMQ” at 5 DAP or 10 DAP or both 5 DAP and 10 DAP (Additional file 1: Table S4, Additional file 1: Table S5), which accords with the above GO enrichment results and suggested again that the early development stages of the hull were the key in determining the hull difference between the two tartary buckwheat cultivars. The expression of 17 identified SCW-related genes, including 7 regulatory genes and 10 enzyme genes, were further verified to be highly similar (r ≥ 0.78) to those observed in RNA-seq data by RT-qPCR analyses (Fig. 10). This indicated the reliability of the transcriptomic data and the identified genes that caused the hull difference between the two tartary buckwheat cultivars. To further verify that the different expression of these identified SCW biosynthesis genes in the early hull development stages was the reason for the hull difference formation between rice- tartary buckwheat and normal tartary buckwheat, the expression of the first-layer regulators (*NST1*, *NST2*, and *SND1*/*NST3*) of SCW biosynthesis was tested in the hull of the other three normal tartary buckwheat cultivars. As shown in Fig. 11, all three genes showed the highest expression in normal tartary buckwheat cultivars at 10 DAP, and the significantly different expression (fold change > 2) between “XMQ” and normal tartary buckwheat cultivars occurred both at both 5 DAP and 10 DAP. This suggested that the different expression of these SCW biosynthesis genes at the early hull development stages was the primary reason the hull difference between the rice-tartary buckwheat and normal tartary buckwheat.

### “In silico” promoter analysis of hub TFs and enzyme genes involved in SCW biosynthesis revealed the presence of binding sites of SCW-related TFs

The SCW-related TF binding cis-elements SNBE (NAC binding) [22] and SMRE (MYB biding) [23] were investigated in the promoter sequences of nine SCW-related TFs (*NST1*, *NST2*, *SND1*/*SNT3*, *MYB54*, *MYB46*/*MYB83*, *MYB103* (2), *C3H14*, and *C3H15*), four cellulose biosynthesis-related enzyme genes (*CESA4*, *CESA7*, *CESA8*, and *XTH22*), eight hemicellulose biosynthesis-related enzyme genes (*IRX8*, *IRX9*, *IRX14-L*, *GXM1*, *GUX5*, *TBL3*, *TBL31*, and *TBL33*) and other 19 identified hub TFs. As results, the SNBE and SMRE cis-elements presented in the promoter sequences of almost all SCW-related TFs and enzyme genes (Table 1). In addition, a high number of SNBE cis-elements were also found in the promoter sequences of the other 19 hub TFs, while there no or very few SMRE cis-elements displayed in these hub TFs (Table 1). Due to ethylene response *EIN3* TF was found in hub TFs and it had been demonstrated playing crucial regulatory role in many developmental processes [24], so we further investigated the EIN3 binding cis-element in promoter sequences of the above genes. As shown in Table 1, the EIN3 binding cis-element appeared in the promoter sequences of 33 out of the 40 genes, in which the highest number of EIN3 binding cis-elements were found in the first-layer NAC regulators of SCW biosynthesis (*NST1*, *NST2*, and *NST3*/*SND1*) (15, 15, and 9) and the xylem vessel formation regulator *XND1* (11), suggesting that the EIN3 might be the direct up-stream regulator of the first-layer NAC regulators of SCW biosynthesis.

**Table 1:** The number of cell wall-related (SNBE and SMRE) and EIN3 binding cis-elements presented in the promoter sequences of the identified hub genes in MEred module

Gene ID	Annotation	SNBE	SMRE	EIN3 motif
FtPinG0000381200.01	NST1	3	1	15
FtPinG0002596000.01	SND1/NST3	2	3	15
FtPinG0007471500.01	NST2	2	2	9
FtPinG0007716800.01	MYB46/83	9	1	1
FtPinG0008420900.01	MYB103	1	2	2
FtPinG0005092500.01	MYB103	4	2	1
FtPinG0004122100.01	MYB52/54	8	9	nd
FtPinG0004517800.01	C3H14	1	3	2
FtPinG0008083100.01	C3H15	7	3	nd
FtPinG0000375900.01	CESA4	2	5	4
FtPinG0003961800.01	CESA7	3	3	4
FtPinG0002305900.01	CESA8	1	4	4
FtPinG0006414500.01	XTH22	1	3	2
FtPinG0003892200.01	IRX9	nd	3	1
FtPinG0006419200.01	IRX14-L	1	3	6
FtPinG0002882400.01	GXM1	3	2	nd
FtPinG0005387700.01	IRX8	3	6	2
FtPinG0008408600.01	GUX5	2	nd	2
FtPinG0007617500.01	TBL3	1	2	2
FtPinG0000963000.01	TBL31	1	2	1
FtPinG0002445000.01	TBL33	nd	3	5
FtPinG0007202700.01	B3 TF	1	1	2
FtPinG0001825500.01	bZIP TF	4	2	3
FtPinG0009370700.01	bZIP TF	2	2	2
FtPinG0001521000.01	DBB TF	9	2	nd
FtPinG0005157600.01	Dof TF	9	0	3
FtPinG0006457000.01	EIN3	7	0	1
FtPinG0000926400.01	ERF	2	1	2
FtPinG0005918800.01	G2-like	3	1	nd
FtPinG0009119800.01	GeBP	2	0	nd
FtPinG0001034500.01	LBD	4	1	2
FtPinG0003417600.01	MYB	6	2	2
FtPinG0004292100.01	XND1	6	2	11
FtPinG0005490300.01	VIN2	5	0	2
FtPinG0006575700.01	Nin-like	2	1	3
FtPinG0007073300.01	RAV	9	0	2
FtPinG0002155700.01	TALE	7	2	9
FtPinG0008730100.01	TALE	7	1	4
FtPinG0007227700.01	WRKY	8	1	7
FtPinG0005108600.01	ZF-HD	5	2	nd

## Discussion

Tartary buckwheat, a highly nutritious medicinal and edible crop, has attracted worldwide attention and gained popularity in the food marketplace. However, the difficult dehulling of normal tartary buckwheat cultivars has severely limited the development of its food processing industry. Fortunately, rice-tartary buckwheat, a very rare and easily dehulled tartary buckwheat, was found in nature [10]. Several recent studies have characterized the genetic and partly physiological mechanisms of the easy dehulling of rice- tartary buckwheat [11–13, 18, 21]. Nevertheless, to date, the detailed physiological, cellular, and especially molecular, mechanisms involved in the easy dehulling of rice-tartary buckwheat remains largely unknown. In this study, we performed comparative cell, physiology, and transcriptome analyses to gain insight into the easy dehulling mechanism of rice-tartary buckwheat and identified the key gene regulatory network that is responsible the easy dehulling of rice-tartary buckwheat.

A manual dehulling analysis found that the hull of rice-tartary buckwheat “XMQ” was brittler than that of normal tartary buckwheat cultivar “JQ”. In many reported brittle culm mutants from *Arabidopsis thaliana*, rice, sorghum and maize, it had been demonstrated that the brittleness is caused by reduced cellulose, and/or hemicellulose, and/or lignin contents [23, 25, 26]. In our study, we found that the cellulose content of “XMQ” was significantly lower than in all determined normal tartary buckwheat cultivars, which was consistent with the previous report [21]. Similarly, obviously lower hemicellulose and lignin contents were also observed in “XMQ” when compared with some normal tartary buckwheat cultivars. These results suggested that the reduced cellulose, hemicellulose and lignin contents of the “XMQ” hull led to its hull brittleness and made it easy to dehull, and the reduced cellulose content might be the major reason. In previous reports, the cellulose content of both rice-tartary buckwheat and normal tartary buckwheat cultivar was found to sustain growth during hull development [21]. In this study, we obtained a similar result concerning the cellulose and hemicellulose contents in “XMQ” and “JQ”. In addition, the significant difference in cellulose and hemicellulose contents between the two cultivars began at 10 DAP and continued to 20 DAP. These indicated that the cellulose and hemicellulose contents of tartary buckwheat hull dynamically change during hull development and that the early hull development stage was the key period during which the difference between rice-tartary buckwheat and normal tartary buckwheat hulls was formed. Recently, several studies found that the hull ditch of rice-tartary buckwheat was thinner than normal tartary buckwheat, which suggested that the thin hull ditch of rice-tartary buckwheat contributed to the easy dehulling property [12, 13, 18]. In our study, we also observed that the hull ditch of “XMQ” was significantly thinner than “JQ” at late stages of hull development (15 and 20 DAP). In addition, we also found that the cell wall thickness of sclerenchyma cells of “XMQ” hull ditch was also obviously thinner than “JQ”. Notably, in brittle culm mutants of *Arabidopsis thaliana*, rice, sorghum and maize, the reduced cellulose, hemicellulose, and lignin contents lead to thinner SCWs than those in the wild type [23, 25, 26]. Therefore, our these findings indicated that the lower hull cellulose and hemicellulose contents also contributed to the thinner hull ditch of rice-tartary buckwheat through reduced the cell wall thickness of the hull sclerenchyma cells.

Consistent with the physiological observations, our transcriptome data also showed that the early hull development stages were the key period in determining the hull difference between rice-tartary buckwheat and normal tartary buckwheat. In *A. thaliana* and some other plants, many regulatory genes (major NAC and MYB TFs) and structural genes of SCW biosynthesis have been functional identified, and the gene regulation network of SCW biosynthesis has also been established [26–31]. In our study, we identified a module with genes showing specific high expression in “JQ” at 10 DAP by using WGCNA for all identified EDGs between “XMQ” and “JQ” hulls at four development stages. In this module, 9 identified hub TFs were found to be the homologs of *A. thaliana* SCW biosynthesis regulation genes, which included the homologs of the first-layer (*NST1*, *NST2*, and *SND1*) [32–34], the second-layer (*MYB46*/*MYB83*) [35, 36] and the third-layer regulators (*MYB54*, *MYB103*, *C3H14*, and *C3H15*) [37, 38]. Furthermore, 24 homologs of the *A. thaliana* SCW biosynthesis structural genes, which catalyzed cellulose, hemicellulose, pectin, and lignin biosynthesis, were also identified in this module [26, 31], and 12 of them were also defined as hub genes. Based on the RNA-seq data, the expression of these identified SCW biosynthesis regulatory and structural genes were significantly higher (fold change > 2) in “JQ” than that in “XMQ” at 5 DAP or 10 DAP or both 5 DAP and 10 DAP, which was further verified by qRT-PCR. In addition, the expression of the first-layer regulators (*NST1*, *NST2*, and *SND1*) was also obviously higher in the other three normal tartary buckwheat cultivars in the early hull development stages. In *Arabidopsis* and other plants, the mutation of the SCW biosynthesis regulatory or structural genes would lead to a severe reduction of cellulose, hemicellulose, or lignin contents and the thin of SCW, and finally caused a collapsed vessel and brittle phenotype [26–38]. Therefore, combining the above-mentioned cellular and physiological data as well as these transcriptome data, our results suggested that the lower expression of SCW biosynthesis regulatory and structural genes in the early development stages of rice-tartary buckwheat hull was the reason for the reduction of cellulose and hemicellulose contents, which further led to the thin hull ditch, the thin cell wall of sclerenchyma cell, the brittle hull of rice-tartary buckwheat, and finally contributes to its easy dehulling.

In *Arabidopsis*, the first-layer NAC and the second-layer MYB regulators of SCW biosynthesis can be bound to the SNBE and SMRE sequences in the promoter sequences of its down-stream target genes and directly activate the expression of target genes [22, 23]. In our study, we found that higher number of SNBE sequence existed in the second-layer regulator (*MYB46*/*MYB83*) and some other hub TFs, while no or fewer number of SNBE sequence in the third-layer regulators and structural genes. In contrast, higher number of SMRE sequence were found in the third-layer regulators and structural genes. In *Arabidopsis*, the *MYB46* and *MYB83* have been demonstrated as the direct targets of the first-layer NAC regulators [35, 36], and the third-layer regulators are the direct targets of the second-layer MYB regulators [23]. Therefore, our results indicated that a conserved gene regulatory network for SCW biosynthesis exists in tartary buckwheat hull. In addition, other identified hub TFs, which had not shown functional characterization involved in SCW biosynthesis in previous studies, might also participate in the regulation of SCW biosynthesis by acting as the direct targets of the first-layer NAC regulators. Notably, the homologs of *EIN3* was identified as hub TF in the SCW biosynthesis module and more *EIN3* binding motifs (15, 15, and 9) [24] were found in the promoter sequences of the first-layer NAC regulators, which implied that the *EIN3* might be the direct up-stream regulatory gene of the first-layer NAC regulators in SCW biosynthesis.

## Conclusions

**Fig. 12:**
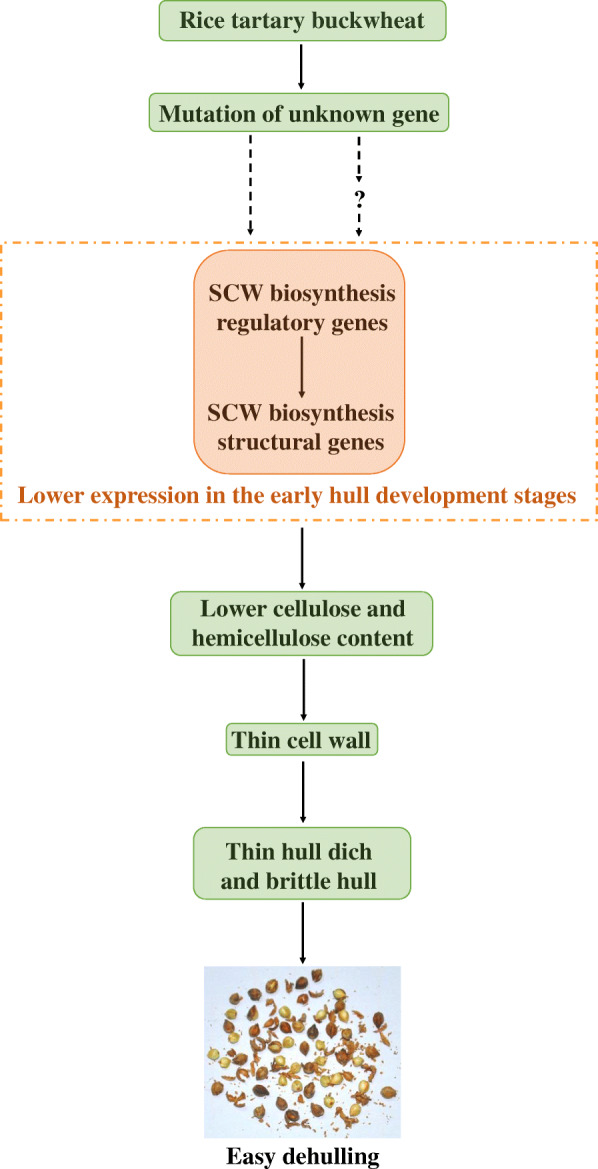
Speculative schematic illustration of the possible easy dehulling mechanism of rice-tartary buckwheat

In the present study, we performed an integrated analysis of the comparative cellular, physiological, transcriptome, and gene coexpression network to investigate the reason that rice-tartary buckwheat is easy to dehull. Our results suggest that the lower expression of SCW biosynthesis regulatory and structural genes in rice-tartary buckwheat hull in the early development stages contribute to its easy dehulling by reducing the content of cell wall chemical components (cellulose and hemicellulose). On this basis, it further led to the thinner cell wall in hull sclerenchyma cells, thinner hull, and lower hull mechanical strength of rice-tartary buckwheat. A schematic that attempted to illustrate the easy dehulling mechanism of rice-tartary buckwheat was drawn based on our results (Fig. 12). These findings helped us better understand the cell, physiology and molecular mechanisms of the underlying easy dehulling formation in rice-tartary buckwheat seeds. Additionally, our data also provided valuable molecular information for the future hull improvement of normal tartary buckwheat cultivar through gene manipulations such as gene expression interference and gene editing.

## Methods

### Plant material and sampling

Five tartary buckwheat cultivars original from our own lab, including the easily dehulled rice-tartary buckwheat (“XMQ”) and four examples of difficult-to-dehulled normal tartary buckwheat (“JQ”, “JJQ”, “PQ” and “CQ”), were used in this study. They were grown in the experimental field of the Research Center of Buckwheat Industry Technology, Guizhou Normal University (Lat. 26°49′ N, 106°58′ E, Alt. 1245 m), China, in spring 2019. Flowers were tagged when they were fully open (finished pollination). For “XMQ” and “JQ”, more than 15,000 flowers were tagged for each cultivar. For the other three cultivars, about 2000 flowers were tagged for each cultivar. Seeds of all five cultivars were collected at 5, 10, 15 and 20 DAP with three biological replicates, respectively. In addition, the fully mature seeds (not tagged) of all five cultivars were also harvested. For transcriptome analysis, the seed hulls of “XMQ” and “JQ” at four differential development stages were stripped on dry ice, immediately frozen in liquid nitrogen and stored at − 80 °C. For paraffin section analysis, the seed samples were immediately soaked in 10% formalin to fix tissues after collection. For transmission electron microscopy analysis, the seed samples were immediately soaked in electron microscope fixative after collection. For physiological analysis, seeds were dried at 60 °C to constant weight, and then the hulls were collected.

### Measurement of cellulose, hemicellulose, and lignin

The dried hulls of mature seeds were used to determine the amounts of cellulose, hemicellulose, and lignin in the five different cultivars. In addition, dynamic accumulation of cellulose and hemicellulose during hull development was investigated using “XMQ” and “JQ” seeds at four different development stages. The measurement of cellulose, hemicellulose and lignin was performed based on the Van Soest method [39].

### Scanning electron microscopy, paraffin section, and transmission electron microscopy analyses

Mature “XMQ” and “JQ” seeds were observed by scanning electron microscopy, which was carried out as previously described [40]. For paraffin section analysis, “XMQ” and “JQ” seeds of four different development stages were used, and the treatment was performed as previously described [40]. The hull ditch thickness of “XMQ” and “JQ” were measured using CaseViewer software based on the paraffin section result. For each sample, 30 points were selected to measure the hull ditch thickness. For transmission electron microscopy analysis, the 15 DAP seeds of “XMQ” and “JQ” were used, and hull ditch was observed by Servicebio Co., Ltd. (Wuhan, China). The cell wall thickness of “XMQ” and “JQ” sclerenchyma cell was measured by using ImageJ software. For each cultivar, 10 cells were selected and 10 points (uniformly distributed on cell) were measured for each cell.

### RNA sequencing, read mapping and DEGs analyses

For RNA-seq, total RNA extraction and library construction for each sample were carried out as described in previously published research [2]. A total of 24 libraries (eight samples with three biological replicates) were sequenced using the BGISEQ-500 system by Huada Gene Technology Co., Ltd. (Shenzhen, China) to generate raw reads. Then the clean reads were obtained by removing the adaptor sequences and low-quality reads using the Trimmomatic (v0.36) [41]. The clean reads were mapped on the tartary buckwheat genome (http://www.mbkbase.org/Pinku1/) using HISAT2 (v2.1.0) with default parameters [1, 42]. The mapped clean reads were further matched to the reference gene sequence of tartary buckwheat by Bowtie2 (v2.2.5) [43], and then RSEM software was used to calculate the gene expression value (FPKM, fragments per kilobase of transcript length per million mapped reads) [44]. Correlation between the biological replicates was determined using the Pearson correlation coefficient (PCC). Hierarchical clustering analysis (HCA) and principal component analysis (PCA) were performed as previously described [45]. The significant DEGs between the samples were identified using the DESeq package based on the threshold of |log2(fold change)| ≥1 and a FDR (false discovery rate) value of < 0.05 [2]. The stage-specific/preferential genes in both cultivars were identified via the stage specificity (SS) scoring algorithm as described previously [45, 46]. We set a SS threshold of 0.5 for detecting stage-specific/preferential genes in both cultivars.

### GO enrichment and gene coexpression network analyses

GO enrichment analysis of stage-specific/preferential genes and DEGs were performed according to the description in Garg et al. [45]. GO enrichment networks were visualized using Cytoscape 2.8.2. For gene coexpression network analysis, WGCNA was carried out based on the Langfelder and Horvath method [47].

### Identification of key modules and genes for the hull difference formation

The key module of the hull difference formation was identified based on comprehensive consideration of the following: (1) the feature of easy dehulling trait (recessive trait), (2) physiological analysis, and (3) PCA analysis of all expression genes. All genes in the key modules were subjected to NR annotation and homology query in the TAIR database of *Arabidopsis thaliana* to identify genes involved in the hull difference formation. The gene regulatory network of the identified key module was visualized using Cytoscape 2.8.2.

### RT-qPCR analysis and correlation verified

Seventeen identified key genes for the hull difference formation were selected to confirm the expression in “XMQ” and “JQ” by qRT-PCR. In addition, the expression of three hub TFs from the 17 selected key genes, which were the first-layer regulators of SCW biosynthesis, were also tested in the hulls of other three normal tartary buckwheat cultivars. qRT-PCR analysis was performed as described by Li et al. [48]. The tartary buckwheat actin7 gene was used as the internal control. The primers used in this study are listed in Additional file 1: Table S6.

### Statistical analysis

Data was statistically analyzed by one-way ANOVA followed by Tukey’s test or Student’s t-test using SPSS 18.0 software. A *P*-value of < 0.05 was identified as a statistically significant difference.

## Supplementary Information

**Additional file 1: Table S1.** Summary statistics of RNA-seq data in different samples for XMQ and JQ. **Table S2.** List of GO enrichment of DEGs between XMQ and JQ hull at four different development stages. **Table S3.** Identified regulatory and structural genes of SCW biosynthesis in MEred module. **Table S4.** The expression value (FPKM) of the identified regulatory and structural genes of SCW biosynthesis and other hub TFs in MEred module. **Table S5.** The fold changes of the identified regulatory and structural genes of SCW biosynthesis and other hub TFs between XMQ and JQ hull at different development stages. **Table S6.** Primers of sequences for qRT-PCR analysis.

**Additional file 2: Figure S1.** Correlation heatmap between transcriptomes of three biological replicates of each sample from XMQ and JQ. **Figure S2.** The number of expressed genes (A) and the proportion of genes expressed at different levels (based on FPKM) (B) in different samples in XMQ and JQ. **Figure S3.** Pearson correlation (A) and Principal component analyses (B) of RNA-seq data from four stages of hull development in XMQ and JQ. **Figure S4.** GO enrichment map (biological process) of preferentially expressed genes at 5 DAP of hull development in XMQ and JQ. **Figure S5.** GO enrichment map (biological process) of preferentially expressed genes at 15 DAP of hull development in XMQ and JQ. **Figure S6.** GO enrichment map (biological process) of preferentially expressed genes at 20 DAP of hull development in XMQ and JQ. **Figure S7.** The number of genes from different TF families showing up- or downregulation in XMQ during seed hull development. **Figure S8.** Module-cellulose and hemicellulose content associations (A) and the genes expression heatmap of the module with higher association with cellulose content (B).

## Data Availability

The datasets supporting the conclusions of this article are included within the article and its additional files. All the raw data from the RNA-seq are available in the Sequencing Read Archive (SRA) of NCBI under the BioProject number PRJNA666523. The short read archives for the RNA-seq data can be found under numbers: SRR12764403, SRR12764404, SRR12764405, SRR12764406, SRR12764407, SRR12764408, SRR12764409, SRR12764410, SRR12764411, SRR12764412, SRR12764413, SRR12764414, SRR12764415, SRR12764416, SRR12764417, SRR12764418, SRR12764419, SRR12764420, SRR12764421, SRR12764422, SRR12764423, SRR12764424.

## Abbreviations

DAP: Days after pollination
WGCNA: Weighted gene coexpression network analysis
DEGs: Differentially expressed genes
FDR: False discovery rate
FPKM: Fragments per kilobase of transcript length per million mapped reads
SCW: Secondary cell wall
qRT-PCR: Quantitative real-time polymerase chain reaction
PCC: Pearson correlation coefficient
HCA: Hierarchical clustering analysis
PCA: Principal component analysis
SS: Stage specificity
GO: Gene ontology
TFs: Transcription factors
