# Supplementary material for: Comparative cellular, physiological and transcriptome analyses reveal the potential easy dehulling mechanism of rice-tartary buckwheat (Fagopyrum Tararicum)
Source: BMC Plant Biol. 2020 Nov 4;20:505. doi: 10.1186/s12870-020-02715-7 (PMC7640676; doi:10.1186/s12870-020-02715-7)
Supplement: Supplementary file 1 — Additional file 1: Table S1. Summary statistics of RNA-seq data in different samples for XMQ and JQ. Table S2. List of GO enrichment of DEGs between XMQ and JQ hull at four different development stages. Table S3. Identified regulatory and structural genes of SCW biosynthesis in MEred module. Table S4. The expression value (FPKM) of the identified regulatory and structural genes of SCW biosynthesis and other hub TFs in MEred module. Table S5. The fold changes of the identified regulatory and structural genes of SCW biosynthesis and other hub TFs between XMQ and JQ hull at different development stages. Table S6. Primers of sequences for qRT-PCR analysis. [file 12870_2020_2715_MOESM1_ESM.zip › Additional file 1-Table S1.docx]

**Table S1.** Summary statistics of RNA-seq data in different samples for XMQ and JQ.

| **JQ** | | | | | | |
| --- | --- | --- | --- | --- | --- | --- |
| Sample  name | Stage/tissue | Replicate | Raw reads | Clean reads | Total Mapping(%) | Uniquely Mapping(%) |
| J5 | 5 DAP hull | R1 | 49079576 | 42911242 | 91.63 | 73.22 |
| J5 | 5 DAP hull | R2 | 50702814 | 43895972 | 91.51 | 73.47 |
| J5 | 5 DAP hull | R3 | 50832418 | 44172788 | 91.06 | 73.39 |
| J10 | 10 DAP hull | R1 | 50634216 | 43419254 | 91.08 | 72.7 |
| J10 | 10 DAP hull | R2 | 50139432 | 43878790 | 91.07 | 73.03 |
| J10 | 10 DAP hull | R3 | 49657633 | 43364582 | 92.04 | 73.18 |
| J15 | 15 DAP hull | R1 | 49273957 | 43014914 | 91.19 | 71.25 |
| J15 | 15 DAP hull | R2 | 51636130 | 41400554 | 85.84 | 64.54 |
| J15 | 15 DAP hull | R3 | 49039563 | 43121570 | 91.87 | 73.12 |
| J20 | 20 DAP hull | R1 | 50225328 | 43157428 | 89.89 | 70.47 |
| J20 | 20 DAP hull | R2 | 49175347 | 42066104 | 90.63 | 70.13 |
| J20 | 20 DAP hull | R3 | 50342588 | 43320402 | 89.2 | 68.48 |
| **XMQ** | | | | | | |
| Sample  name | Stage/tissue | Replicate | Raw reads | Clean reads | Total Mapping(%) | Uniquely Mapping(%) |
| X5 | 5 DAP hull | R1 | 47326734 | 42783630 | 92.02 | 73.07 |
| X5 | 5 DAP hull | R2 | 47628755 | 42892160 | 92.49 | 73.32 |
| X5 | 5 DAP hull | R3 | 48306588 | 43101038 | 92.2 | 73.05 |
| X10 | 10 DAP hull | R1 | 49597596 | 44668478 | 92.74 | 73.34 |
| X10 | 10 DAP hull | R2 | 48775779 | 44053302 | 92.17 | 73.18 |
| X10 | 10 DAP hull | R3 | 47025794 | 42976824 | 92.39 | 73.14 |
| X15 | 15 DAP hull | R1 | 49593827 | 43845276 | 91.9 | 71.77 |
| X15 | 15 DAP hull | R2 | 49265681 | 43428840 | 91.66 | 71.95 |
| X15 | 15 DAP hull | R3 | 46395793 | 42833240 | 92.55 | 71.77 |
| X20 | 20 DAP hull | R1 | 47149435 | 42880148 | 91.67 | 72.59 |
| X20 | 20 DAP hull | R2 | 48826570 | 43021184 | 90.32 | 70.77 |
| X20 | 20 DAP hull | R3 | 49974938 | 44904840 | 90.65 | 71.87 |
