# Supplementary material for: Comparative cellular, physiological and transcriptome analyses reveal the potential easy dehulling mechanism of rice-tartary buckwheat (Fagopyrum Tararicum)
Source: BMC Plant Biol. 2020 Nov 4;20:505. doi: 10.1186/s12870-020-02715-7 (PMC7640676; doi:10.1186/s12870-020-02715-7)
Supplement: Supplementary file 1 — Additional file 1: Table S1. Summary statistics of RNA-seq data in different samples for XMQ and JQ. Table S2. List of GO enrichment of DEGs between XMQ and JQ hull at four different development stages. Table S3. Identified regulatory and structural genes of SCW biosynthesis in MEred module. Table S4. The expression value (FPKM) of the identified regulatory and structural genes of SCW biosynthesis and other hub TFs in MEred module. Table S5. The fold changes of the identified regulatory and structural genes of SCW biosynthesis and other hub TFs between XMQ and JQ hull at different development stages. Table S6. Primers of sequences for qRT-PCR analysis. [file 12870_2020_2715_MOESM1_ESM.zip › Additional file 1-Table S3.docx]

| **Gene ID** | ***Arabidopsis* homologous** | **Annotation** | **Function** |
| --- | --- | --- | --- |
| **1^st^ layer regulatory factors** | | | |
| FtPinG0000381200.01 | NST1/AT2G46770.1 | NAC transcription factor | Activates MYB46/83 and lower-level MYBs; induces cellulose, xylan, and lignin biosynthesis; regulating secondary wall thickening. |
| FtPinG0007471500.01 | NST2/AT3G61910.1 | NAC transcription factor | Functional redundancy with NST1 |
| FtPinG0002596000.01 | SND1/NST3/AT1G32770.1 | NAC transcription factor | Positively regulate fiber SCW thickening and cellulose and lignin biosynthesis. |
| **2^st^ layer regulatory factor** | | | |
| FtPinG0007716800.01 | MYB46/MYB83/AT3G08500.1 | MYB transcription factor | Activates lower-level MYBs; induces cellulose, xylan, and lignin biosynthesis. |
| **3^st^ layer regulatory factors** | | | |
| FtPinG0008420900.01 | MYB103/AT1G63910.1 | MYB transcription factor | Positively regulate fiber SCW thickening; induces secondary wall-related cellulose and lignin biosynthesis. |
| FtPinG0005092500.01 | MYB103/AT1G63910.1 | MYB transcription factor | Positively regulate fiber SCW thickening; induces secondary wall-related cellulose and lignin biosynthesis. |
| FtPinG0004122100.01 | MYB54/AT1G73410.1 | MYB transcription factor | Regulate hemicellulose biosynthesis. |
| FtPinG0008083100.01 | C3H14/AT1G66810.1 | CCCH zinc finger protein | Regulate secondary wall thickening |
| FtPinG0004517800.01 | C3H15/ AT1G68200.1 | CCCH zinc finger protein | Regulate secondary wall thickening |
| **Enzyme genes for cellulose biosynthesis** | | | |
| FtPinG0000375900.01 | CESA4/IRX5/AT5G44030.1 | Cellulose synthase | Catalyzing secondary wall cellulose biosynthesis |
| FtPinG0003961800.01 | CESA7/IRX3/AT5G17420.1 | Cellulose synthase | Catalyzing secondary wall cellulose biosynthesis |
| FtPinG0002305900.01 | CESA8/IRX1/AT4G18780.1 | Cellulose synthase | Catalyzing secondary wall cellulose biosynthesis |
| FtPinG0006695400.01 | COBL4/IRX6/AT5G15630.1 | COBL protein, similar to phytochelatin synthetase | Catalyzing secondary wall cellulose biosynthesis |
| FtPinG0004459500.01 | GDPDL3/AT4G26690.1 | Glycerophosphoryl diester phosphodiesterase-like protein | Regulate cell wall cellulose accumulation and pectin linking |
| FtPinG0006414500.01 | XTH22/AT5G57560.1 | Cell wall-modifying enzyme | Catalyse covalent cross-linking between cellulose and cello-oligosaccharide |
| **Enzyme genes for hemicellulose biosynthesis** | | | |
| FtPinG0006406600.01 | UXS2/ AUD1/AT3G62830.1 | UDP-glucuronic acid decarboxylase | Catalyzing hemicellulose and pectin biosynthesis |
| FtPinG0005327100.01 | UXS5/AT3G46440.1 | UDP-glucuronic acid decarboxylase | Catalyzing hemicellulose and pectin biosynthesis |
| FtPinG0003892200.01 | IRX9/AT2G37090.1 | GT43 family glycosyltransferase | Catalyzing hemicellulose glucuronoxylan biosynthesis |
| FtPinG0001270000.01 | IRX9-L/I9H/AT1G27600.1 | GT43 family glycosyltransferase | Catalyzing hemicellulose glucuronoxylan biosynthesis |
| FtPinG0006419200.01 | IRX14-L/I14H/AT5G67230.1 | GT43 family glycosyltransferases | Hemicellulose glucuronoxylan biosynthesis |
| FtPinG0002882400.01 | GXM1/AT1G09610.1 | Glucuronoxylan 4-O-methyltransferase-like protein | Catalyzing 4-O-methylation of hemicellulose glucuronoxylan |
| FtPinG0005387700.01 | GAUT12/IRX8/AT5G54690.1 | Galacturonosyltransferase | Catalyzing hemicellulose xylan and pectin biosynthesis |
| FtPinG0008408600.01 | GUX5/AT1G08990.1 | Glucuronyltransferases | Catalyzing hemicellulose glucuronoxylan biosynthesis |
| FtPinG0007224400.01 | UXT1/AT2G28315.1 | Nucleotide/sugar transporter | Catalyzing xylan biosynthesis |
| FtPinG0007617500.01 | TBL3/AT5G01360.1 | DUF231-containing  O-acetyltransferases | Catalyzing hemicellulose xylan acetylation |
| FtPinG0000963000.01 | TBL31/AT1G73140.1 | DUF231-containing  O-acetyltransferases | Catalyzing hemicellulose xylan acetylation |
| FtPinG0002445000.01 | TBL33/AT2G40320.1 | DUF231-containing  O-acetyltransferases | Catalyzing hemicellulose xylan acetylation |
| **Enzyme genes for pectin biosynthesis** | | | |
| FtPinG0000034500.01 | GATL2/AT3G50760.1 | Galacturonosyltransferase | Catalyzing pectin biosynthesis |
| **Enzyme genes for lignin biosynthesis** | | | |
| FtPinG0009593000.01 | F5H/AT4G36220.1 | Ferulate 5-hydroxylase | Involved in lignin biosynthesis |
| FtPinG0007978200.01 | CCoAOMT1/AT4G34050.1 | Caffeoyl coenzyme A  O-methyltransferase | Involved in lignin biosynthesis |
| FtPinG0007156500.01 | LAC4/IRX12/AT2G38080.1 | Laccase | Involved in lignin biosynthesis |
| FtPinG0004216800.01 | LAC17/ AT5G60020.1 | Laccase | Catalyzing G lignin biosynthesis |
| FtPinG0006420900.01 | PRX52/AT5G05340.1 | Peroxidase | Involved in lignin biosynthesis |
