# Supplementary material for: Comparative cellular, physiological and transcriptome analyses reveal the potential easy dehulling mechanism of rice-tartary buckwheat (Fagopyrum Tararicum)
Source: BMC Plant Biol. 2020 Nov 4;20:505. doi: 10.1186/s12870-020-02715-7 (PMC7640676; doi:10.1186/s12870-020-02715-7)
Supplement: Supplementary file 1 — Additional file 1: Table S1. Summary statistics of RNA-seq data in different samples for XMQ and JQ. Table S2. List of GO enrichment of DEGs between XMQ and JQ hull at four different development stages. Table S3. Identified regulatory and structural genes of SCW biosynthesis in MEred module. Table S4. The expression value (FPKM) of the identified regulatory and structural genes of SCW biosynthesis and other hub TFs in MEred module. Table S5. The fold changes of the identified regulatory and structural genes of SCW biosynthesis and other hub TFs between XMQ and JQ hull at different development stages. Table S6. Primers of sequences for qRT-PCR analysis. [file 12870_2020_2715_MOESM1_ESM.zip › Additional file 1-Table S5.docx]

| **Gene Name** | **Gene ID** | **X5 vs J5** | | **X10 vs J10** | | **X15 vs J15** | | **X20 vs J20** | |
| --- | --- | --- | --- | --- | --- | --- | --- | --- | --- |
|  |  | **Log2FC** | **P value** | **Log2FC** | **P value** | **Log2FC** | **P value** | **Log2FC** | **P value** |
| **Regulatory factors of secondary cell wall biosynthesis** | | | | | | | | | |
| NST1 | FtPinG0000381200.01 | NA | NA | 1.44 | 5.86E-56 | NA | NA | NA | NA |
| NST2 | FtPinG0007471500.01 | 2.55 | 1.71E-06 | 1.36 | 4.21E-162 | NA | NA | NA | NA |
| SND1/NST3 | FtPinG0002596000.01 | 1.06 | 5.45E-64 | NA | NA | NA | NA | NA | NA |
| MYB46/MYB83 | FtPinG0007716800.01 | 1.18 | 1.68E-40 | NA | NA | NA | NA | NA | NA |
| MYB103 | FtPinG0008420900.01 | 1.90 | 8.96E-25 | 1.49 | 0 | NA | NA | NA | NA |
| MYB103 | FtPinG0005092500.01 | NA | NA | 1.74 | 6.19E-172 | NA | NA | -1.56 | 1.97E-08 |
| MYB54 | FtPinG0004122100.01 | 2.10 | 2.50E-12 | 1.18 | 1.43E-60 | NA | NA | NA | NA |
| C3H14 | FtPinG0008083100.01 | 1.47 | 2.91E-46 | NA | NA | NA | NA | -4.34 | 2.17E-35 |
| C3H15 | FtPinG0004517800.01 | 1.42 | 4.57E-42 | NA | NA | NA | NA | -3.09 | 3.82E-05 |
| **Regulatory factors of xylem vessel formation** | | | | | | | | | |
| XND1 | FtPinG0004292100.01 | NA | NA | 1.92 | 1.24E-105 | NA | NA | NA | NA |
| VNI2 | FtPinG0005490300.01 | 2.98 | 6.63E-15 | 3.59 | 3.61E-25 | NA | NA | NA | NA |
| **Enzyme genes for cellulose biosynthesis** | | | | | | | | | |
| CESA4 | FtPinG0000375900.01 | 1.15 | 0 | NA | NA | NA | NA | NA | NA |
| CESA7 | FtPinG0003961800.01 | 1.55 | 6.50E-260 | NA | NA | NA | NA | NA | NA |
| CESA8 | FtPinG0002305900.01 | 1.71 | 0 | NA | NA | NA | NA | NA | NA |
| IRX6 | FtPinG0006695400.01 | 1.48 | 2.30E-268 | NA | NA | NA | NA | NA | NA |
| GDPDL3 | FtPinG0004459500.01 | NA | NA | NA | NA | NA | NA | -1.75 | 0 |
| **Enzyme genes for hemicellulose biosynthesis** | | | | | | | | | |
| UXS2 | FtPinG0006406600.01 | NA | NA | 1.19 | 0 | NA | NA | NA | NA |
| UXS5 | FtPinG0005327100.01 | NA | NA | 1.04 | 0 | NA | NA | -1.00 | 3.25E-146 |
| IRX9 | FtPinG0003892200.01 | 1.13 | 2.95E-145 | NA | NA | NA | NA | NA | NA |
| IRX9-L | FtPinG0001270000.01 | NA | NA | NA | NA | NA | NA | -1.36 | 5.27E-06 |
| IRX14-L | FtPinG0006419200.01 | 1.87 | 1.50E-83 | NA | NA | NA | NA | NA | NA |
| GXM1 | FtPinG0002882400.01 | NA | NA | 1.11 | 7.67E-217 | NA | NA | -1.00 | 5.51E-36 |
| IRX8 | FtPinG0005387700.01 | 1.02 | 2.66E-24 | NA | NA | NA | NA | NA | NA |
| GUX5 | FtPinG0008408600.01 | 2.21 | 3.64E-20 | 1.37 | 3.50E-291 | NA | NA | NA | NA |
| UXT1 | FtPinG0007224400.01 | 1.12 | 8.54E-189 | NA | NA | NA | NA | 1.04 | 4.53E-165 |
| TBL3 | FtPinG0007617500.01 | 1.15 | 0 | NA | NA | NA | NA | NA | NA |
| TBL31 | FtPinG0000963000.01 | 1.13 | 0.00032 | NA | NA | NA | NA | 1.56 | 1.03E-06 |
| TBL33 | FtPinG0002445000.01 | 1.92 | 5.24-08 | 1.03 | 9.83E-25 | NA | NA | NA | NA |
| **Enzyme genes for lignin biosynthesis** | | | | | | | | | |
| GATL2 | FtPinG0000034500.01 | NA | NA | NA | NA | NA | NA | 1.46 | 7.89E-245 |
| CCoAOMT1 | FtPinG0007978200.01 | NA | NA | 1.72 | 0 | NA | NA | NA | NA |
| IRX12 | FtPinG0007156500.01 | NA | NA | 2.55 | 1.16E-263 | 1.43 | 2.44E-50 | -1.29 | 6.89E-10 |
| LAC17 | FtPinG0004216800.01 | 1.13 | 5.42E-54 | NA | NA | NA | NA | -3.37 | 2.81E-11 |
| PRX52 | FtPinG0006420900.01 | 2.26 | 2.59E-142 | 1.50 | 1.64E-183 | NA | NA | 2.49 | 3.95E-191 |
| **Other hub TFs** | | | | | | | | | |
| B3 TF | FtPinG0007202700.01 | NA | NA | 2.38 | 6.42E-16 | NA | NA | NA | NA |
| bZIP | FtPinG0001825500.01 | NA | NA | 1.70 | 6.13E-15 | NA | NA | NA | NA |
| bZIP | FtPinG0009370700.01 | NA | NA | 3.01 | 0.0045 | NA | NA | NA | NA |
| DBB TF | FtPinG0001521000.01 | NA | NA | 2.33 | 0.00013 | NA | NA | NA | NA |
| Dof TF | FtPinG0005157600.01 | NA | NA | 1.36 | 0.00034 | NA | NA | NA | NA |
| EIN3 | FtPinG0006457000.01 | NA | NA | 2.53 | 6.22E-180 | NA | NA | -1.68 | 6.31E-44 |
| ERF71 | FtPinG0000926400.01 | NA | NA | 2.01 | 6.55E-61 | 1.44 | 7.35E-31 | NA | NA |
| G2-like TF | FtPinG0005918800.01 | NA | NA | 1.20 | 3.49E-31 | NA | NA | 1.05 | 2.90E-06 |
| GeBP TF | FtPinG0009119800.01 | NA | NA | 1.10 | 0 | NA | NA | NA | NA |
| LBD | FtPinG0001034500.01 | -1.13 | 5.70E-15 | NA | NA | NA | NA | NA | NA |
| MYB TF | FtPinG0003417600.01 | NA | NA | NA | NA | NA | NA | -1.70 | 8.83E-23 |
| Nin-like TF | FtPinG0006575700.01 | 1.16 | 8.68E-08 | 1.79 | 3.06E-26 | NA | NA | NA | NA |
| RAV | FtPinG0007073300.01 | NA | NA | 1.03 | 4.58E-39 | NA | NA | NA | NA |
| TALE TF | FtPinG0002155700.01 | 1.62 | 5.64E-25 | NA | NA | NA | NA | NA | NA |
| TALE TF | FtPinG0008730100.01 | NA | NA | 1.50 | 5.62E-17 | NA | NA | NA | NA |
| WRKY TF | FtPinG0007227700.01 | NA | NA | 3.66 | 3.91E-14 | NA | NA | -5.56 | 1.35E-08 |
| ZF-HD TF | FtPinG0005108600.01 | NA | NA | 1.26 | 2.56E-82 | NA | NA | 1.41 | 3.51E-07 |
