# Supplementary figures and images for: Comparative cellular, physiological and transcriptome analyses reveal the potential easy dehulling mechanism of rice-tartary buckwheat (Fagopyrum Tararicum)
Source: BMC Plant Biol. 2020 Nov 4;20:505. doi: 10.1186/s12870-020-02715-7 (PMC7640676; doi:10.1186/s12870-020-02715-7)

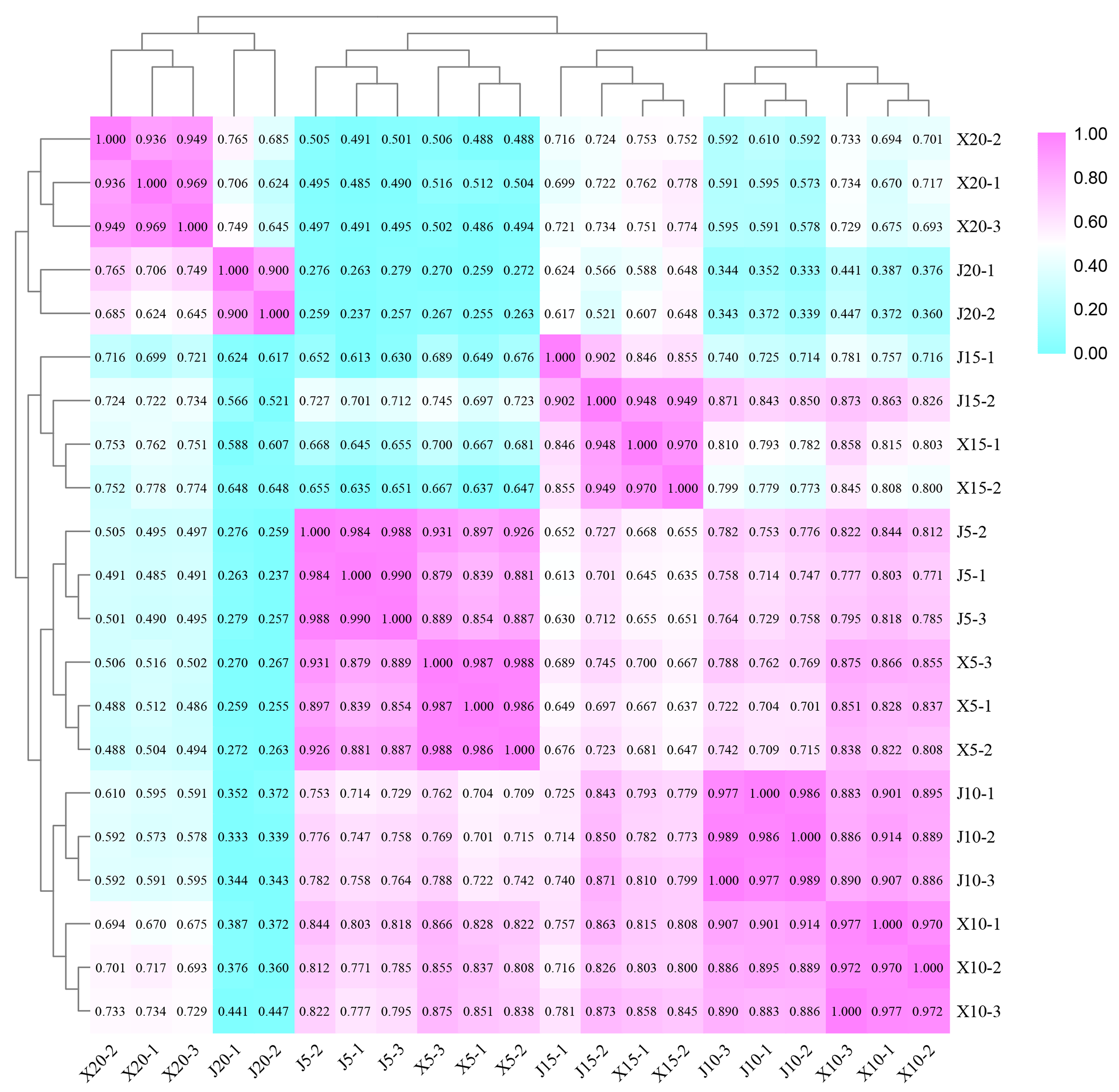

Supplement: Supplementary file 2 — Additional file 2: Figure S1. Correlation heatmap between transcriptomes of three biological replicates of each sample from XMQ and JQ. Figure S2. The number of expressed genes (A) and the proportion of genes expressed at different levels (based on FPKM) (B) in different samples in XMQ and JQ. Figure S3. Pearson correlation (A) and Principal component analyses (B) of RNA-seq data from four stages of hull development in XMQ and JQ. Figure S4. GO enrichment map (biological process) of preferentially expressed genes at 5 DAP of hull development in XMQ and JQ. Figure S5. GO enrichment map (biological process) of preferentially expressed genes at 15 DAP of hull development in XMQ and JQ. Figure S6. GO enrichment map (biological process) of preferentially expressed genes at 20 DAP of hull development in XMQ and JQ. Figure S7. The number of genes from different TF families showing up- or downregulation in XMQ during seed hull development. Figure S8. Module-cellulose and hemicellulose content associations (A) and the genes expression heatmap of the module with higher association with cellulose content (B). [file 12870_2020_2715_MOESM2_ESM.zip › Additional file 2-Figure S1.tif]

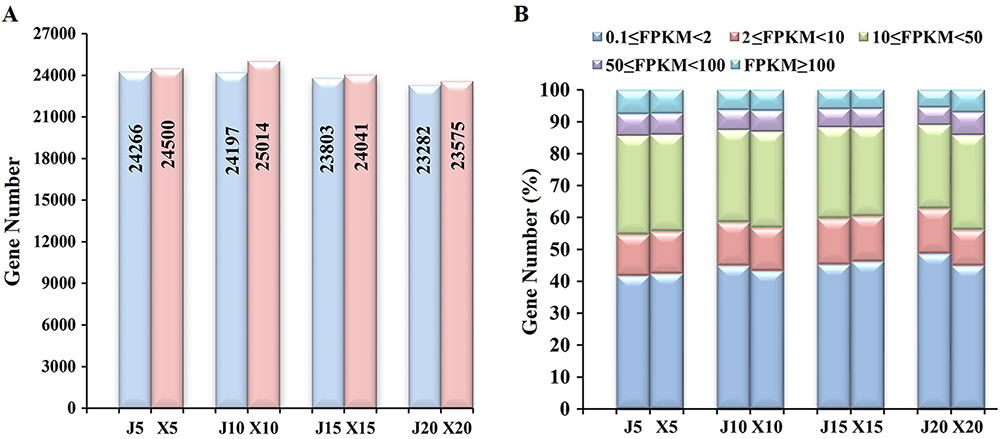

Supplement: Supplementary file 2 — Additional file 2: Figure S1. Correlation heatmap between transcriptomes of three biological replicates of each sample from XMQ and JQ. Figure S2. The number of expressed genes (A) and the proportion of genes expressed at different levels (based on FPKM) (B) in different samples in XMQ and JQ. Figure S3. Pearson correlation (A) and Principal component analyses (B) of RNA-seq data from four stages of hull development in XMQ and JQ. Figure S4. GO enrichment map (biological process) of preferentially expressed genes at 5 DAP of hull development in XMQ and JQ. Figure S5. GO enrichment map (biological process) of preferentially expressed genes at 15 DAP of hull development in XMQ and JQ. Figure S6. GO enrichment map (biological process) of preferentially expressed genes at 20 DAP of hull development in XMQ and JQ. Figure S7. The number of genes from different TF families showing up- or downregulation in XMQ during seed hull development. Figure S8. Module-cellulose and hemicellulose content associations (A) and the genes expression heatmap of the module with higher association with cellulose content (B). [file 12870_2020_2715_MOESM2_ESM.zip › Additional file 2-Figure S2.tif]

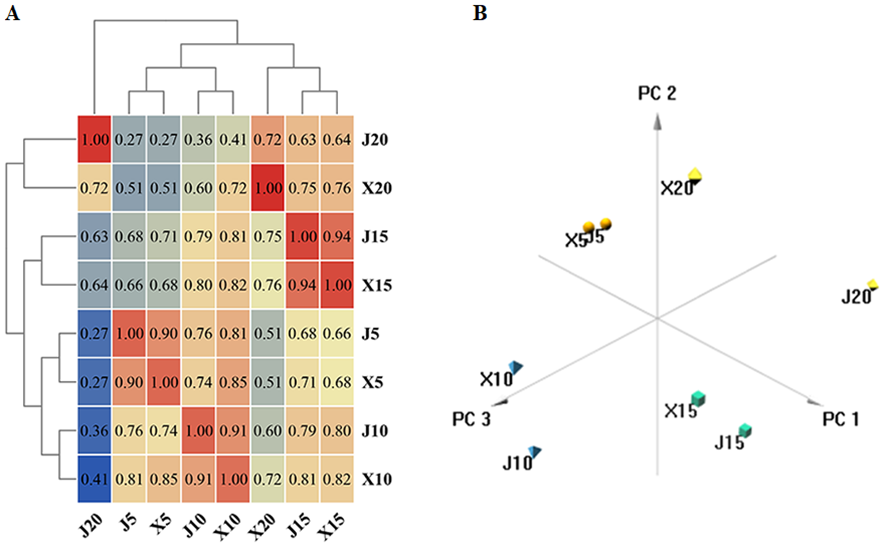

Supplement: Supplementary file 2 — Additional file 2: Figure S1. Correlation heatmap between transcriptomes of three biological replicates of each sample from XMQ and JQ. Figure S2. The number of expressed genes (A) and the proportion of genes expressed at different levels (based on FPKM) (B) in different samples in XMQ and JQ. Figure S3. Pearson correlation (A) and Principal component analyses (B) of RNA-seq data from four stages of hull development in XMQ and JQ. Figure S4. GO enrichment map (biological process) of preferentially expressed genes at 5 DAP of hull development in XMQ and JQ. Figure S5. GO enrichment map (biological process) of preferentially expressed genes at 15 DAP of hull development in XMQ and JQ. Figure S6. GO enrichment map (biological process) of preferentially expressed genes at 20 DAP of hull development in XMQ and JQ. Figure S7. The number of genes from different TF families showing up- or downregulation in XMQ during seed hull development. Figure S8. Module-cellulose and hemicellulose content associations (A) and the genes expression heatmap of the module with higher association with cellulose content (B). [file 12870_2020_2715_MOESM2_ESM.zip › Additional file 2-Figure S3.tif]

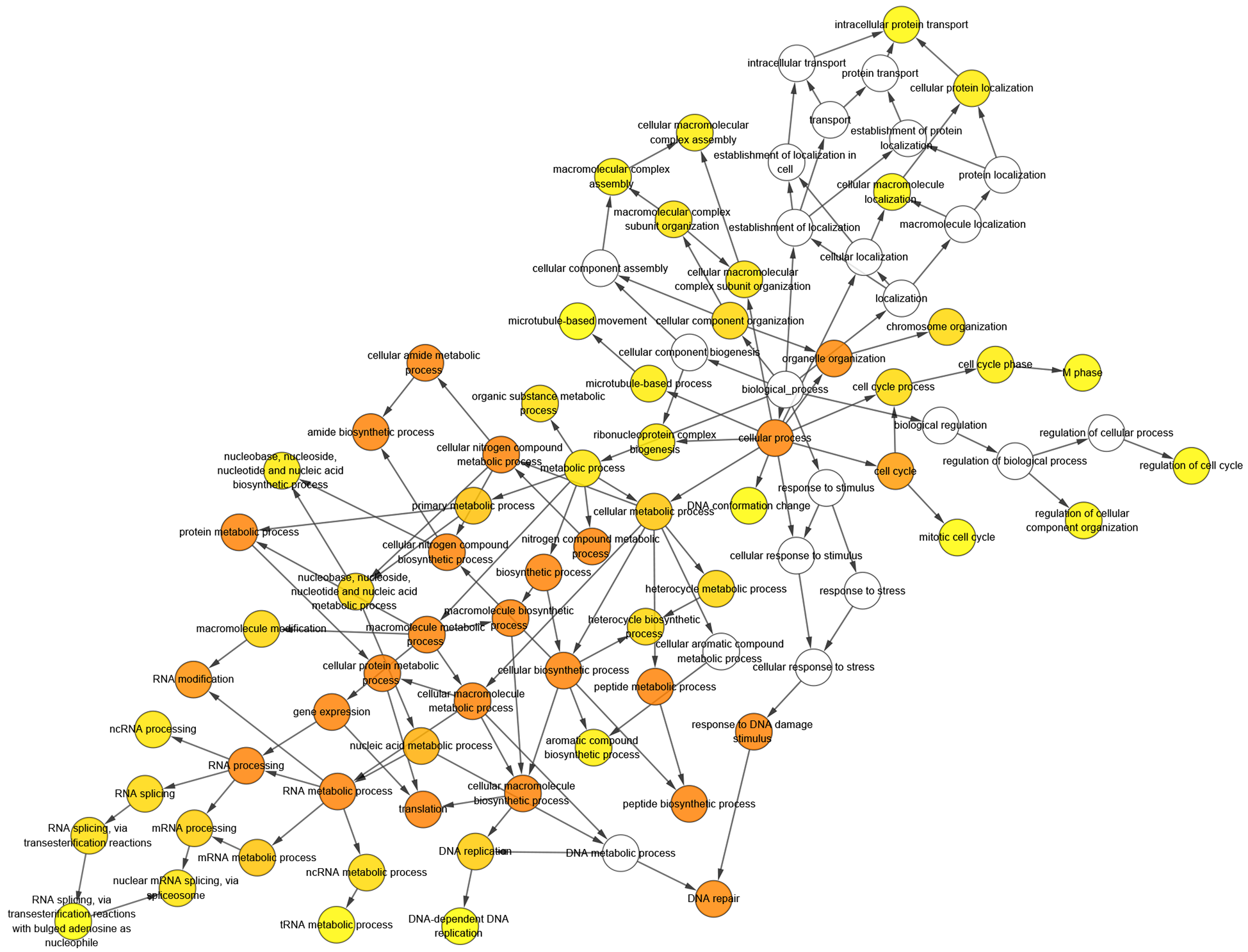

Supplement: Supplementary file 2 — Additional file 2: Figure S1. Correlation heatmap between transcriptomes of three biological replicates of each sample from XMQ and JQ. Figure S2. The number of expressed genes (A) and the proportion of genes expressed at different levels (based on FPKM) (B) in different samples in XMQ and JQ. Figure S3. Pearson correlation (A) and Principal component analyses (B) of RNA-seq data from four stages of hull development in XMQ and JQ. Figure S4. GO enrichment map (biological process) of preferentially expressed genes at 5 DAP of hull development in XMQ and JQ. Figure S5. GO enrichment map (biological process) of preferentially expressed genes at 15 DAP of hull development in XMQ and JQ. Figure S6. GO enrichment map (biological process) of preferentially expressed genes at 20 DAP of hull development in XMQ and JQ. Figure S7. The number of genes from different TF families showing up- or downregulation in XMQ during seed hull development. Figure S8. Module-cellulose and hemicellulose content associations (A) and the genes expression heatmap of the module with higher association with cellulose content (B). [file 12870_2020_2715_MOESM2_ESM.zip › Additional file 2-Figure S4.tif]

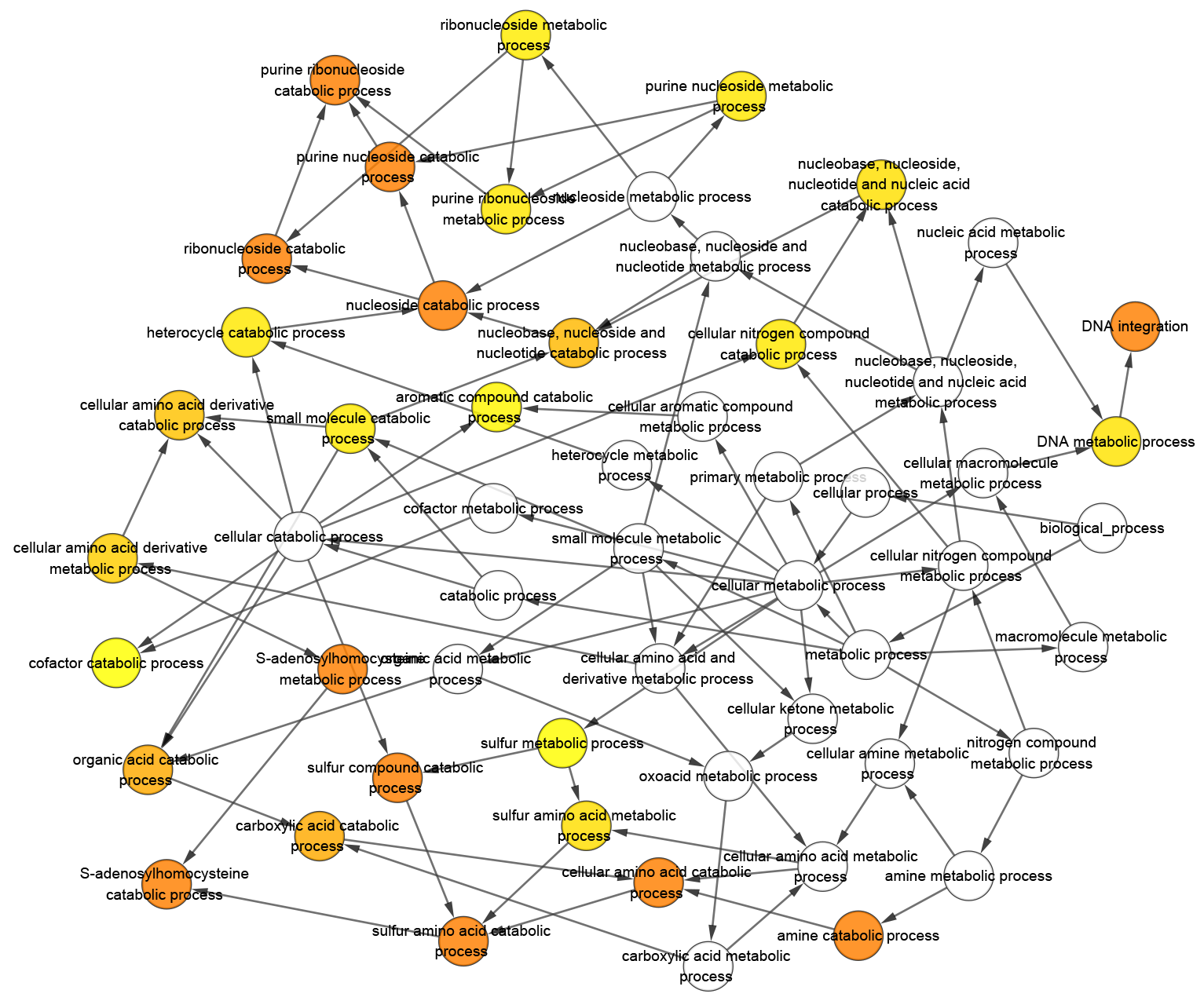

Supplement: Supplementary file 2 — Additional file 2: Figure S1. Correlation heatmap between transcriptomes of three biological replicates of each sample from XMQ and JQ. Figure S2. The number of expressed genes (A) and the proportion of genes expressed at different levels (based on FPKM) (B) in different samples in XMQ and JQ. Figure S3. Pearson correlation (A) and Principal component analyses (B) of RNA-seq data from four stages of hull development in XMQ and JQ. Figure S4. GO enrichment map (biological process) of preferentially expressed genes at 5 DAP of hull development in XMQ and JQ. Figure S5. GO enrichment map (biological process) of preferentially expressed genes at 15 DAP of hull development in XMQ and JQ. Figure S6. GO enrichment map (biological process) of preferentially expressed genes at 20 DAP of hull development in XMQ and JQ. Figure S7. The number of genes from different TF families showing up- or downregulation in XMQ during seed hull development. Figure S8. Module-cellulose and hemicellulose content associations (A) and the genes expression heatmap of the module with higher association with cellulose content (B). [file 12870_2020_2715_MOESM2_ESM.zip › Additional file 2-Figure S5.tif]

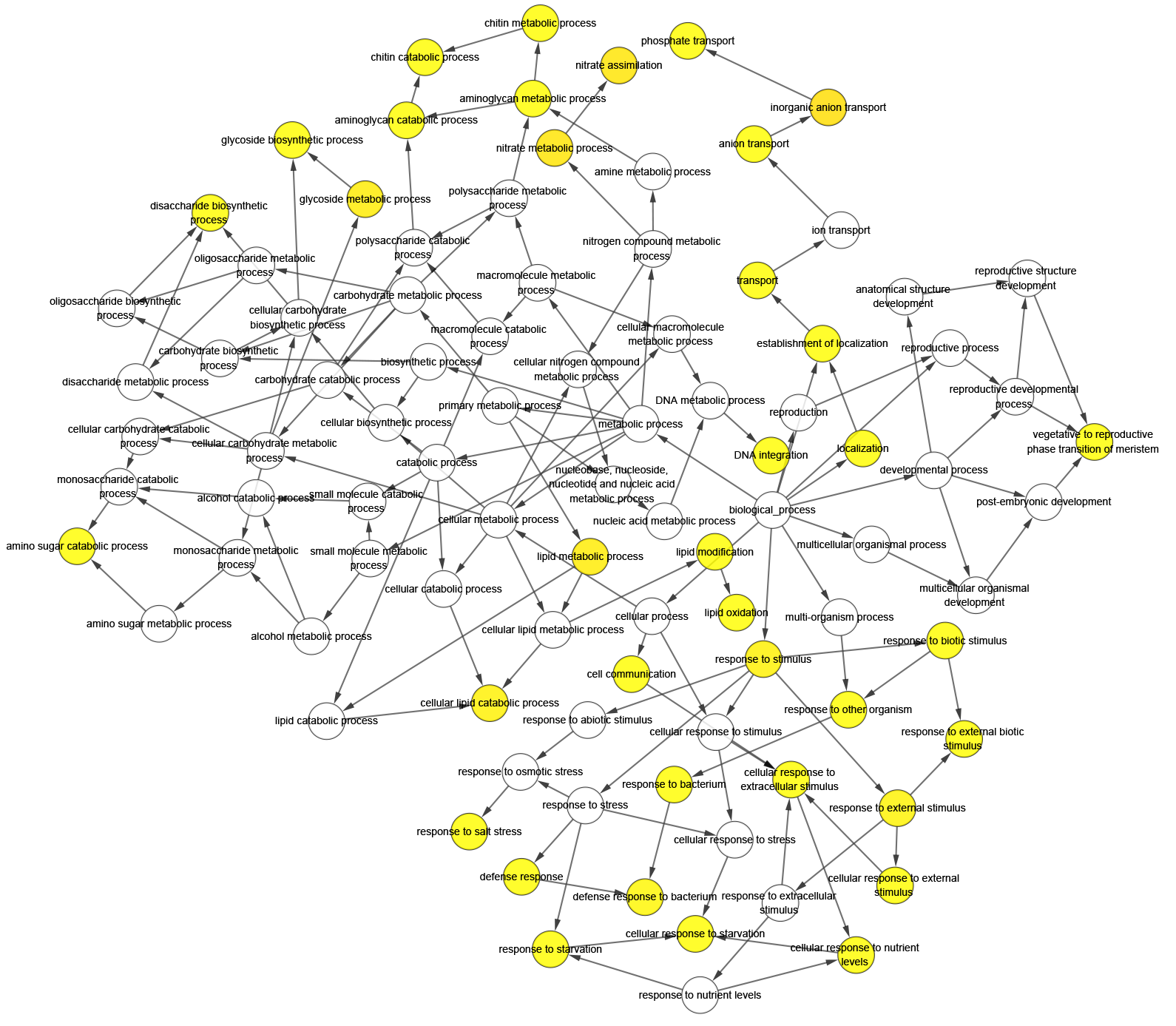

Supplement: Supplementary file 2 — Additional file 2: Figure S1. Correlation heatmap between transcriptomes of three biological replicates of each sample from XMQ and JQ. Figure S2. The number of expressed genes (A) and the proportion of genes expressed at different levels (based on FPKM) (B) in different samples in XMQ and JQ. Figure S3. Pearson correlation (A) and Principal component analyses (B) of RNA-seq data from four stages of hull development in XMQ and JQ. Figure S4. GO enrichment map (biological process) of preferentially expressed genes at 5 DAP of hull development in XMQ and JQ. Figure S5. GO enrichment map (biological process) of preferentially expressed genes at 15 DAP of hull development in XMQ and JQ. Figure S6. GO enrichment map (biological process) of preferentially expressed genes at 20 DAP of hull development in XMQ and JQ. Figure S7. The number of genes from different TF families showing up- or downregulation in XMQ during seed hull development. Figure S8. Module-cellulose and hemicellulose content associations (A) and the genes expression heatmap of the module with higher association with cellulose content (B). [file 12870_2020_2715_MOESM2_ESM.zip › Additional file 2-Figure S6.tif]

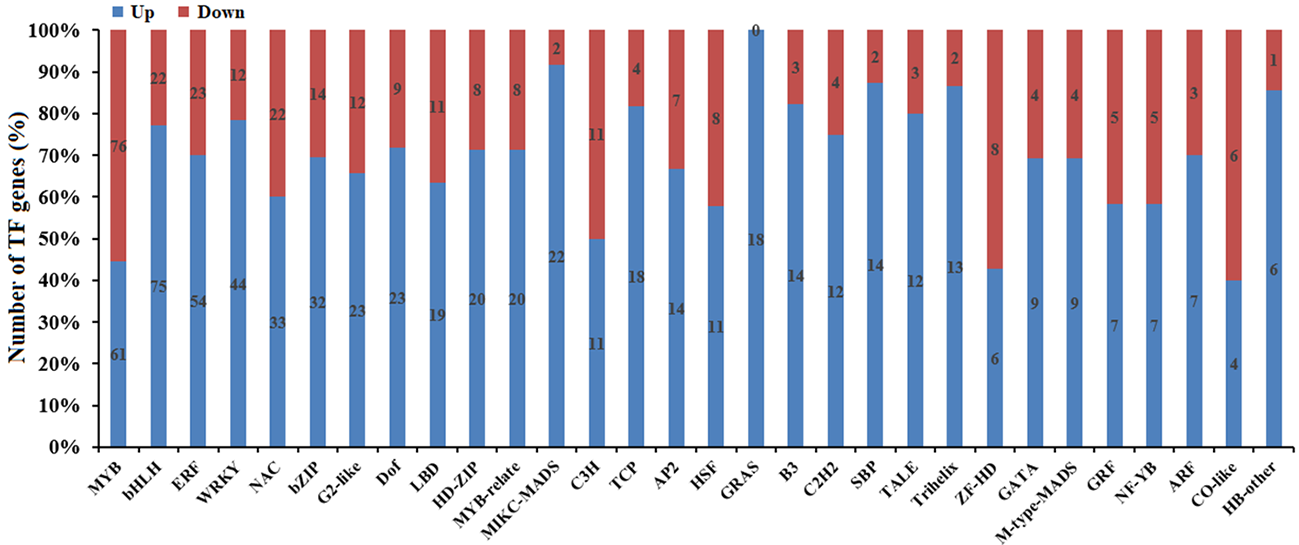

Supplement: Supplementary file 2 — Additional file 2: Figure S1. Correlation heatmap between transcriptomes of three biological replicates of each sample from XMQ and JQ. Figure S2. The number of expressed genes (A) and the proportion of genes expressed at different levels (based on FPKM) (B) in different samples in XMQ and JQ. Figure S3. Pearson correlation (A) and Principal component analyses (B) of RNA-seq data from four stages of hull development in XMQ and JQ. Figure S4. GO enrichment map (biological process) of preferentially expressed genes at 5 DAP of hull development in XMQ and JQ. Figure S5. GO enrichment map (biological process) of preferentially expressed genes at 15 DAP of hull development in XMQ and JQ. Figure S6. GO enrichment map (biological process) of preferentially expressed genes at 20 DAP of hull development in XMQ and JQ. Figure S7. The number of genes from different TF families showing up- or downregulation in XMQ during seed hull development. Figure S8. Module-cellulose and hemicellulose content associations (A) and the genes expression heatmap of the module with higher association with cellulose content (B). [file 12870_2020_2715_MOESM2_ESM.zip › Additional file 2-Figure S7.tif]

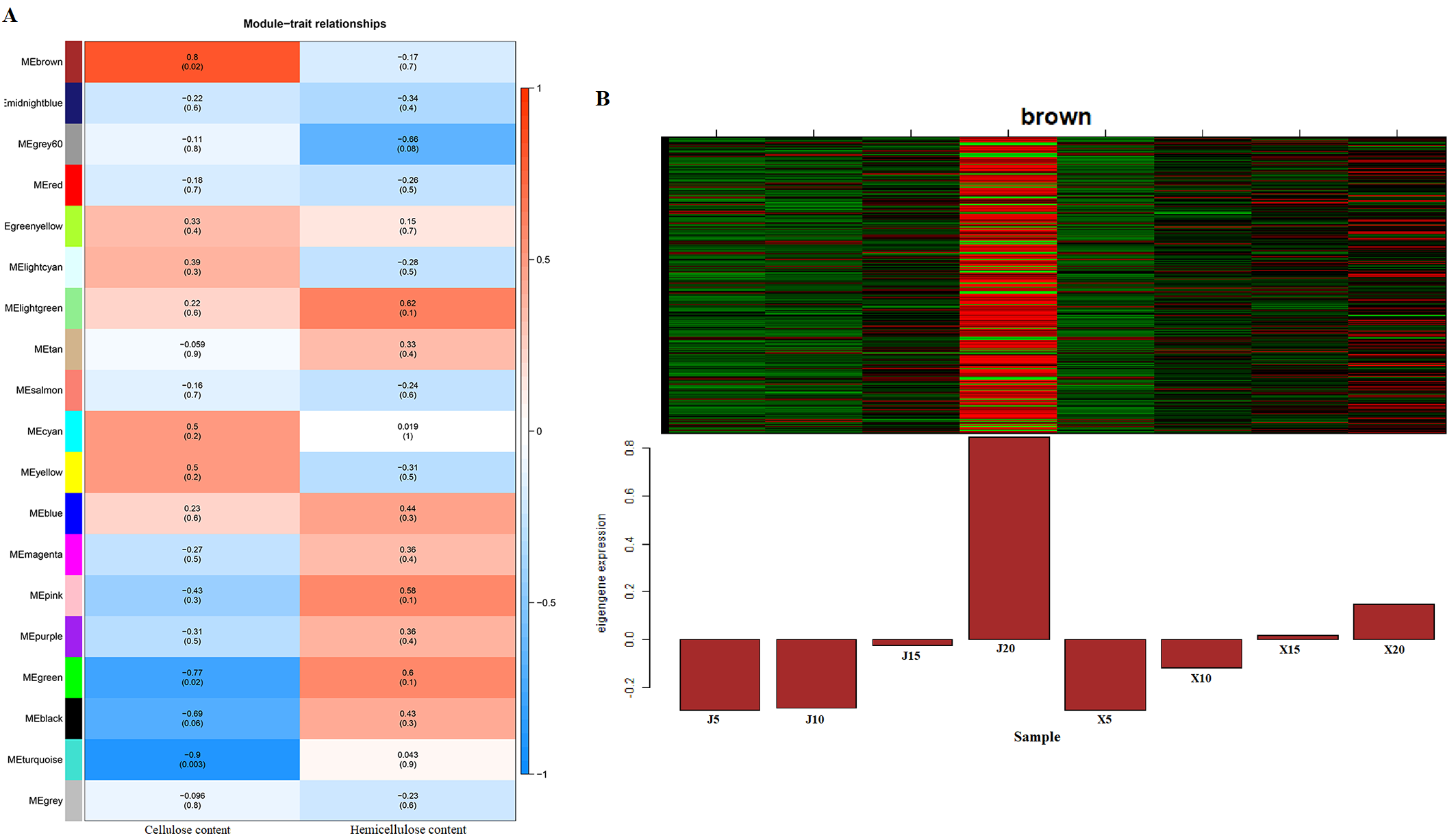

Supplement: Supplementary file 2 — Additional file 2: Figure S1. Correlation heatmap between transcriptomes of three biological replicates of each sample from XMQ and JQ. Figure S2. The number of expressed genes (A) and the proportion of genes expressed at different levels (based on FPKM) (B) in different samples in XMQ and JQ. Figure S3. Pearson correlation (A) and Principal component analyses (B) of RNA-seq data from four stages of hull development in XMQ and JQ. Figure S4. GO enrichment map (biological process) of preferentially expressed genes at 5 DAP of hull development in XMQ and JQ. Figure S5. GO enrichment map (biological process) of preferentially expressed genes at 15 DAP of hull development in XMQ and JQ. Figure S6. GO enrichment map (biological process) of preferentially expressed genes at 20 DAP of hull development in XMQ and JQ. Figure S7. The number of genes from different TF families showing up- or downregulation in XMQ during seed hull development. Figure S8. Module-cellulose and hemicellulose content associations (A) and the genes expression heatmap of the module with higher association with cellulose content (B). [file 12870_2020_2715_MOESM2_ESM.zip › Additional file 2-Figure S8.tif]
